# Voice register in Mon: experiments in production and perception

**DOI:** 10.1515/phon-2024-0047

**Published:** 2025-05-28

**Authors:** Sireemas Maspong, Patrick McCormick, James Kirby

**Affiliations:** Institute for Phonetics and Speech Processing, LMU Munich, Munich, Germany; Center of Excellence in Southeast Asian Linguistics, Faculty of Arts, Chulalongkorn University, Bangkok, Thailand; Center for Southeast Asian Studies, Kyoto University, Kyoto, Japan

**Keywords:** Mon, phonation, register, FPCA, speech perception

## Abstract

*Register* is a two-way contrast realized through a bundle of phonetic properties which may include phonation type, vowel quality, and differences in pitch. Mon, an Austroasiatic language spoken in Myanmar and Thailand, is often described as a prototypical register language. However, reports differ as to which acoustic properties of register are dominant or even present in Mon, and no studies have investigated the extent to which they cue the register contrast in perception. A functional principal component analysis of acoustic and electroglottographic data from seventeen speakers of Burma Mon varieties shows that registers are acoustically differentiated primarily by covarying differences in fundamental frequency (f0) and voice quality. The results of a forced-choice identification study show that listeners are also sensitive to these phonetic properties in perception, but that f0 was the most robust cue to the register contrast. Individual variation is observed in both production and perception, but there is not a straightforward correlation between the two at the individual level. Our analysis suggests that although fundamental frequency is a highly salient cue to register in Burma Mon, it is likely a manifestation of a more general laryngeal configuration rather than a specific acoustic target.

## 1 Introduction

*Register* is a phonological contrast typically characterized by one or more phonetic properties including voice quality, pitch, and vowel quality. It is commonly observed in Austroasiatic languages and other language families across Mainland Southeast Asia (Brunelle and Kirby 2016; Brunelle and Tạ 2021). However, the manifestation and relative importance of these acoustic properties may vary across register languages.

Mon, an Austroasiatic language spoken in Myanmar and Thailand, has been a significant focus in the exploration of register contrast. Mon has a prototypical two-way register contrast between what are traditionally described as ‘head’ and ‘chest’ registers distinguished by primarily by differences in phonation type (Diffloth 1984, 1985; Shorto 1967). However, instrumental studies of Mon register have revealed a more complex picture. Although some studies have found support for the importance of phonation type (Abramson et al. 2015), others have argued for a more prominent role of f0 in distinguishing Mon registers (Lee 1983; Thongkum 1987; Thongkum 1990). Additionally, vowel quality has been suggested as an emerging distinguishing property of Mon register, at least in certain varieties (Behr 2013, 2018).

While several production studies have investigated the Mon register contrast, there remains a noticeable absence of perception studies in the existing literature. The sole perceptual study conducted by Abramson et al. (2015) was as a forced choice perceptual test, wherein listeners were tasked with identifying natural recordings of lexical items produced by native Mon speakers. It therefore did not elucidate the specific cues used for distinguishing registers.

In this paper, we have two main goals. The first goal is to contribute to the understanding of how register contrasts are produced by explicitly considering the extent to which the various acoustic correlates of Mon register co-vary. The second goal is to conduct a perception experiment that systematically manipulates potential acoustic cues to Mon registers. Through this dual approach, we intend to provide a more comprehensive understanding of register contrast in Mon, and to contribute to the phonetic study of register more generally.

To preview our findings, the results of our production study reveal that, at least in the varieties of Burma Mon we have investigated, register is realized through co-varying differences in f0 and voice quality. In terms of perception, we found that listeners relied predominantly on f0 differences to distinguish between registers, but showed some sensitivity to voice and vowel quality when f0 was ambiguous. These findings suggest that the acoustic manifestation of register in Mon is most probably the result of a generally lax laryngeal configuration, rather than explicitly targeting vocal fold tension.

In the remainder of this Introduction, we provide some further background on the phonetic implementation of register contrasts (Section 1.1) as well as on the Mon language (Section 1.2). In Section 2, we analyze acoustic and electroglottographic data from 17 Mon speakers using functional principle component analysis, and in Section 3, we present the results of a perception study designed to assess the weights assigned to different cues to register. In Section 4, we discuss the implications of our findings for our understanding of how register is implemented, both in Mon and more generally.

### 1.1 Register in Southeast Asian languages

Register (sometimes ‘voice register’) is a phonological contrast commonly observed in Austroasiatic and Austronesian languages of Southeast Asia. Here, we focus on the typical case of a binary register contrast, although more complex systems are also attested (e.g., Di Canio 2009). Table 1 summarizes the attested phonetic correlates of register contrasts. Most commonly, register manifests though some combination of voice quality, pitch, vowel quality, and duration, sometimes accompanied by differences in aspiration and voicing of onset consonants.

**Table 1:** Attested phonetic properties of register (Brunelle and Tạ 2021).

High register (also tense, clear, head, or first register)	Low register (also lax, breathy, chest, or second register)
Higher pitch	Lower pitch
Tense/modal voice	Lax/breathy voice
Lower vowels (esp. at their beginning)	Higher vowels (esp. at their beginning)
More peripheral vowels	More centralized vowels
Shorter VOT	Longer VOT
Shorter vowels	Longer vowels

However, there exists considerable diversity in the phonetic realization of register. Not all register languages exhibit each property outlined in Table 1, and moreover these properties may vary in their salience across languages. For instance, *pace* Henderson (1952), in some languages pitch seems to be the dominant acoustic correlate, as observed for example in Kuai (Abramson et al. 2004; Lau-Preechathammarach 2023) or Eastern Cham (Brunelle 2005). In other languages, vowel quality differences are more pronounced, as observed in Chanthaburi Khmer (Wayland and Jongman 2001), Southern Yi (Kuang and Cui 2018), Chru (Brunelle et al. 2020), or Chrau (Tạ et al. 2022). Interestingly, voice quality is rarely identified as the primary correlate of register contrast in phonetic studies, despite the emphasis on its importance for register distinctions made in earlier literature. Mon stands out as one of the few languages where voice quality has been identified as the primary correlate to register, as discussed further in Section 1.2.

As we have just seen, register contrasts are often discussed in terms of ‘primary’ and ‘secondary’ phonetic properties, suggesting that the ‘primary’ feature may be targeted by speakers and most salient to listeners, while ‘secondary’ features may be consequences or side-effects of the production of the primary feature. For example, in their study of a Thai Mon variety spoken in Ban Nakhonchum, Ratchaburi Province, Abramson et al. (2015) propose that voice quality serves as the primary correlate of Mon register, with other properties, particularly f0, considered as automatic consequences of the difference in laryngeal control for phonation types.

In addition, the phonetic realization of register, like other phonological contrasts, can vary and evolve over time (Brunelle et al. 2020, 2022, 2024). For example, some varieties of the Austroasiatic language Khmu have been reported to realize low register syllables with a lax or breathy voice quality (Premsrirat 2001), while other varieties seem to encode the contrast primarily or entirely on the basis of f0 (Abramson et al. 2007; Kirby et al. 2022; Svantesson and House 2006). Indeed, several influential models of tono- and registrogenesis, such as those of Huffman (1976a) and Thurgood (2002), posit that f0-based contrasts first pass through a stage in which voice quality is contrastive (but see Brunelle and Tạ 2021). It is thus entirely possible that different varieties of Mon signal the register contrast with different combinations or weightings of acoustic properties (see e.g., Thongkum 1987). Having a better picture of the ways in which different languages and varieties realize register contrasts will help us better assess and refine our models of how tone and register develop and evolve.

### 1.2 Mon and its register contrast

Mon (ISO-693-3: mnw) is an Austroasiatic language spoken primarily by a population of nearly a million people in the southern regions of Myanmar and the western and central areas of Thailand (Jenny 2015). Mon dialects can be broadly grouped under two main types: Burma Mon and Thai Mon (McCormick and Jenny 2013). Burma Mon is spoken in the southern regions of Myanmar and the western areas of Thailand, while Thai Mon is spoken in the central areas of Thailand. Burma Mon speakers are typically bilingual or fluent in Burmese, while Thai Mon speakers are typically dominant in Thai. Both dialects share cognates, but a significant number of lexical items have been influenced by the dominant languages of the respective countries (Huffman 1976b). Despite these lexical differences, the phonological differences between the two dialects are minimal, although there are important differences in phonetic realization, particular in terms of the vowels (Behr 2018; Huffman 1990). As this paper specifically focuses on Burma Mon, unless otherwise specified, the term ‘Mon’ can be taken as referring to Burma Mon.

The consonant inventory of Mon, as documented by Shorto (1962), is outlined in Table 2. Mon stops do not exhibit contrastive voicing, but they are contrastive for aspiration at all places of articulation, except for the glottal stop. Sonorants, with the exception of /ŋ r j/, do contrast for voicing. Some work (e.g., Jenny 2015) treats the voiceless sonorants as preaspirated. Additionally, the language has preglottalized voiced plosives at the labial and alveolar places of articulation.

**Table 2:** Consonant inventory of Burma Mon.

	Labial	Alveolar	Palatal	Velar	Glottal
Stops	p	t	c	k	ʔ
	p^h^	t^h^	c^h^	k^h^	
	ʔb	ʔd			
Fricatives		s	(ʃ)		h
Nasals	m	n	ɲ	ŋ	
	m̥	n̥	ɲ̊		
Laterals		l			
		l̥			
Rhotics		r			
Glides	w		j		
	w̥				

Mon has a rich and complex vowel inventory. While treatments differ in many details, Burma Mon can be analyzed as contrasting nine monophthongs /i e ɛ a ɤ u o ɔ ɑ/, with /ə/ occurring in minor syllables. Analyses of the diphthongs are more divergent, in part due to the complex distribution of contextual on- and off-glides (Huffman 1990; Shorto 1966), and in part due to a lack of clarity over whether certain vowel-coda (and vowel-register) combinations constitute systematic or accidental gaps. For an overview of some of the issues involved, see Diffloth (1984), esp. chapter 2; Huffman (1990), Jenny (2015), and Shorto (1966).

As in many other register languages, the register contrast in Mon derives from a historical onset voicing contrast: high register corresponds to proto-voiceless onsets, while low register corresponds to proto-voiced onsets. In contemporary Mon, there are no systematic restrictions between register and onset type, i.e., both voiced and voiceless obstruents and sonorants are attested with both registers; however, there do exist certain biases and accidental gaps, e.g., the lack of /ʔd-/ onsets in the low register. In Mon, the register contrast is referred to as a difference between “light” (/sa/) *versus* “heavy” (/sɤ̤ŋ/) syllables, while Western literature variously refers to the registers as “head” *versus* “chest,” “clear” *versus* “breathy,” or “modal” *versus* “breathy.” In this paper, we opt for the terms “high” and “low” to emphasize the abstract nature of the contrast, without evoking any specific phonetic characteristics.

Phonetic descriptions of register in Mon vary considerably. Descriptions from the 1960–1980s describe the contrast as primarily based on phonation type (Diffloth 1984, 1985; Shorto 1967). Shorto (1967) goes so far as to state that “[p]itch difference as an exponent of register is lacking” (1967, p. 246), and that the earlier description by Low (1837, p. 44) of Mon having a tone system was mistaken. However, this view was challenged by subsequent acoustic analyses, primarily of Thai Mon varieties, which observed clear pitch differences between high and low register words (Behr 2013; Lee 1983; Thongkum 1987, 1990). Although some studies also noted differences in spectral energy, suggesting phonation type differences across registers accompanying the pitch differences (Behr 2013; Thongkum 1987), others failed to find such differences (Lee 1983). Diffloth (1982, 1984) reported that although pitch is typically associated with register contrast in Mon, pitch differences were only present in word-by-word elicitation, and the differences were absent in more naturalistic speech, where only phonation type differences remained audible.

The most extensive phonetic study of Mon register to date was conducted by Abramson et al. (2015), who analyzed both acoustic and electroglottographic (EGG) data in a Thai Mon variety. They observed a systematic difference in phonation type across registers, as evidenced by spectral tilt measures, Harmonic-to-Noise Ratio (HNR), Cepstral Peak Prominence (CPP), and Closed-Quotient (CQ) of EGG signals. As a result, they proposed that phonation type is the primary acoustic property signifying Mon register. In contrast, although f0 exhibited significant differences across registers, the effect size was small (approximately 7 Hz). They interpreted this finding as f0 being an automatic consequence of phonation type rather than a property of register controlled by Mon speakers.

Finally, small differences in vowel quality between registers have also been documented for some Mon varieties (Abramson et al. 2015; Behr 2018; Thongkum 1987). However, the difference in vowel quality across registers is not systematic, as the difference is statistically significant only for some vowels (Behr 2018; Thongkum 1987). Some scholars interpret these small differences as evidence that Mon is restructuring its register contrast into a more complex vowel system (Behr 2018), akin to Khmer, while others have proposed that vowel quality does not play a significant role in distinguishing registers in Mon (Abramson et al. 2015).

Despite the considerable body of work on the acoustics of Mon register, the relevance of these cues in perception has not been explored. The sole published perception study (Abramson et al. 2015) involved a forced choice task in which listeners were asked to identify recordings of lexical items with different registers as produced by Mon native speakers, without any manipulation of acoustic cues. While this test validated that Mon listeners can perceive register differences produced by native speakers, it did not investigate which cues Mon speakers use to distinguish registers. Therefore, there is a need for a perception test aimed at identifying the cues used by Mon speakers to distinguish registers.

### 1.3 Research questions

In this paper, we address the following research questions:

(1)

a.

**Table:** 

What is the acoustic realization of register in Mon?

b.

**Table:** 

To what extent do the acoustic correlates of register covary?

c.

**Table:** 

Is there any individual variation in the production of register?

(2)

a.

**Table:** 

What perceptual cues do Mon speakers use to distinguish between high and low register?

b.

**Table:** 

Is there any individual variation in perception?

(3)

**Table:** 

Is there any correlation between the prominence of acoustic properties in production and their salience in perception?

In order to answer these research questions, we conducted both production and perception experiments with speakers of Burma Mon varieties. Both experiments took place at the Mon Lighting Center (Provincial Children and Youth Learning Center) in Samut Sakhon Province, Thailand, in October 2023. All data and associated scripts are available as part of this article’s accompanying OSF archive (https://osf.io/cpz37/?view_only=fcc92d29bce746daa0805b7587a9b274).

## 2 Production experiment

### 2.1 Methods

#### 2.1.1 Participants and data collection

**Figure 1:**
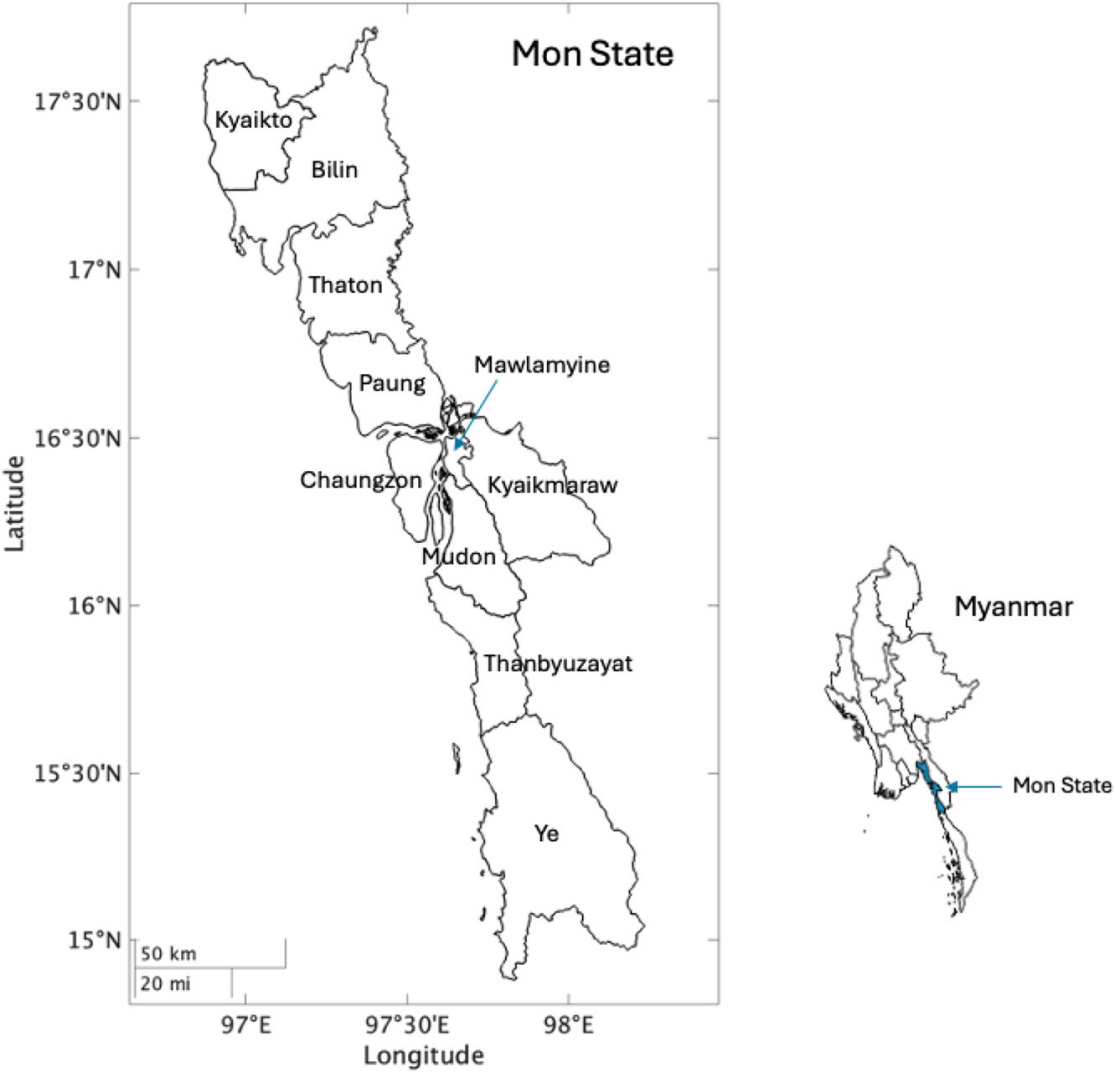
Map of townships in Mon state by the authors using Mon State Village Tract Boundaries MIMU v9.4 (Myanmar Information Management Unit 2023).

We collected acoustic and electroglottographic (EGG) recordings from 17 Burma Mon speakers (6 females and 11 males). They were born between 1970 and 2002 (21–53 years old at the time of the experiment). Participants were from different townships in Mon state: 7 speakers from Paung township, 3 speakers from Mudon township, 2 speakers from Thanbyuzayat township, 2 speakers from Ye township and 1 speaker from Chaungzon (see Figure 1). All participants were native speakers of Mon, with Mon being their primary language. Additionally, they were fluent in Burmese, and all possessed at least a basic conversational proficiency in Central Thai.

During the recording sessions, participants were presented with target words in Mon script, accompanied by corresponding Burmese translations, on a laptop screen. Speakers were instructed to produce the target words within a frame sentence, as shown in 2.1.1. Each target word was repeated three times, except for one speaker (M01) who provided two repetitions and another speaker (F05) who provided only one repetition.

(4)

**Table:** 

ʔare̤ __ ʔoa mo̤ik kɤ̤ʔ hɑm
word __ 1sg want to say
‘I want to say the word __’

The target words consisted of attested Mon monosyllabic words or polysyllabic words with target syllables in final position. The word list was verified with native Mon speakers. Target words and syllables were selected to contain all possible combinations of coronal and velar stop and nasal onsets, as well as high and low registers. We excluded words with voiceless sonorants to limit the duration of the task. We sought a balance between target words occurring in open and closed syllables, with nasal, stop, /h/, and /ʔ/ codas. While prioritizing words with an /a/ nucleus, in cases where such words were not available, we opted for substitutes containing other non-high vowels such as /e ɤ o oi ɛa ɔ ɑ/. It is noteworthy that the vowels /o/ and /ɔ/ in open syllables exhibit variation in vowel height across speakers, regardless of register. For example, some speakers raised /o/ to /o̝/ and /ɔ/ to /ɔ̝/. We therefore analyzed /o/ and /ɔ/ in open syllables separately from those in closed syllables. The final list included 55 words (see Appendix A).

Recordings were made with the SpeechRecorder software (Draxler and Jänsch 2004). The audio signal was captured direct to disk at a sampling rate of 44.1 kHz through a USB digital audio interface connected to a head-mounted microphone. Simultaneously, the EGG signal was captured via a EGG-D800 device from Laryngograph Ltd. The recording sessions took place in a quiet room at the data collection site.

#### 2.1.2 Annotation

All recordings were reviewed by research assistants to identify speech errors, assess recording quality, and verify the presence of the intended target words. Of the 2,640 initial recordings, 21 were excluded due to speech errors. The remaining 2,619 audio recordings were force aligned using the MAUS language-independent model (Schiel 1999). Subsequently, the TextGrids generated by MAUS were manually corrected as needed using Praat (Boersma and Weenink 2020).

After segment intervals were identified, additional annotations were made to mark the onset of voicing and the release of onset closure of the target words. Initially, the onset of voicing was annotated based on the Harmonic-to-Noise Ratio (HNR) for the 0–500 Hz frequency range during the intervals between the target onset and vowel. The HNR values were extracted using PraatSauce (Kirby 2018). The onset of voicing was identified as the first window where the HNR exceeded zero, which served as an indicator of glottal vibration. The annotations of voicing onset were manually reviewed and corrected as needed. Subsequently, the release of onset closure was manually annotated. We did not observe any tokens with pre-voiced onsets where voicing cessation occurred before the release of closure.

#### 2.1.3 Acoustic measurements

In this paper, we focus our analysis on seven acoustic parameters: Voice Onset Time (VOT), spectral center of gravity (CoG) of the release burst, fundamental frequency (f0), the first and second formants (F1 and F2), the corrected difference between the first harmonics and the harmonics at the third formant (H1*-A3*), and Cepstral Peak Prominence (CPP). These measurements were chosen because they have been found to correlate with phonation type and register contrasts in various languages, such as Khmu Rawk (Abramson et al. 2007), Nakhonchum Mon (Abramson et al. 2015), Northern Raglai (Brunelle et al. 2022), Chong (Di Canio 2009), Gujarat, White Hmong (Esposito 2012), Jalapa Mazatec (Garellek and Keating 2011), and Ngãi Giao Chrau (Tạ et al. 2022), among others.

Voice Onset Time (VOT) was determined by calculating the time difference between the annotated closure release and the onset of voicing. Additionally, we computed the Center of Gravity (CoG) of the voiceless stop burst. Following the methodology outlined in Chodroff and Wilson (2014), the recordings were resampled at 16 kHz, pre-emphasized, and high-pass filtered at 200 Hz. For each burst, we generated a smoothed spectrum by averaging the squared amplitudes of five 64-point Fast Fourier Transform (FFT) spectra obtained from 3 ms Hamming windows with a 1 ms time step. The first window was centered at the annotated closure release. If the lag between the annotated closure release and the onset of voicing was shorter than the duration required for five windows, we adjusted the number of spectra to fit within this interval. CoG was then calculated from the smoothed spectra using the formula:

(1) $$\text{CoG}=\frac{\overset{N}{\underset{i=1}{\sum }}f_{i}\cdot A_{i}}{\overset{N}{\underset{i=1}{\sum }}A_{i}}$$

Here, *f*_*i*_ represents the frequency of the *i*th spectral component, *A*_*i*_ represents the amplitude of the *i*th spectral component at frequency *f*_*i*_, and *N* represents the total number of frequency bins in the spectrum.

In addition to VOT and CoG, acoustic measurements were extracted from the annotated files using PraatSauce (Kirby 2018), a set of Praat scripts for extracting spectral measures modeled after VoiceSauce (Shue et al. 2011). Measurements were made every millisecond over the entire recording using a 25 ms analysis window. Fundamental frequency (f0) was estimated using Praat’s autocorrelation method, with a speaker-specific f0 range determined according to the approach proposed by De Looze (2010). Initially, a broad f0 range of 75–400 Hz was utilized for the first pass. Subsequently, the first (*Q*1) and third (*Q*3) quartiles of f0 values for each speaker were calculated. In the second round of f0 tracking, the f0 floor for each participant was set to *Q*1 × 0.75, while the f0 ceiling was set to *Q*3 × 1.5.

Formant frequencies (F1, F2, and F3) were estimated using the Burg LPC algorithm with a tenpole filter and a Gaussian-like analysis window. The formant ceiling was set to 5,000 Hz for male speakers and 5,500 Hz for female speakers. We also measure the H1*-A3*, the difference between the amplitude of the first harmonic (H1) and the most prominent harmonic of the third formant (A3), corrected for the effects of vocal tract resonance using the method of Iseli and Alwan (2004). H1*-A3* has been shown to correlate with the open quotient of the glottis (Bickley 1982) and the velocity of vocal fold closure (Stevens 1977). A larger difference between the first harmonics (H1) and the higher harmonics (e.g., A3) suggests a breathier voice quality. Cepstral Peak Prominence (CPP) is a measure of the periodicity of vocal fold vibrations (jitter, shimmer). CPP was calculated according to the method described by Hillenbrand et al. (1994) as implemented in PraatSauce. Higher CPP values have been found to correlate with a more modal voice quality (Garellek 2019; Garellek and Esposito 2023; Seyfarth and Garellek 2018).

Spurious measurements of f0, F1, F2, and F3 were removed if they deviated by three standard deviations from the mean computed for each combination of speaker, vowel, and register. This process resulted in the removal of approximately 1 % of data points. Following this, H1*-A3* measures at time points where f0 and F1/F3 had been excluded were also discarded.

#### 2.1.4 EGG signal

The EGG signals were processed using *praatdet* (Kirby 2020), a Praat script designed for EGG signal processing. The EGG signals underwent high-pass filtering with a 40 Hz pass frequency and a 20 Hz smoothing cutoff. Additionally, we specified the f0 minimum and maximum thresholds to be the speaker-specific range used for f0 tracking of acoustic signals (see Section 2.1.3). The smoothing window size parameter was set to *k* = 20 preceding and following each period. The smoothed signals were used to calculate the open quotient (OQ) using the method proposed by Howard (1995), with the amplitude threshold set to 3:7 of the EGG cycle’s peak-to-peak amplitude.

#### 2.1.5 Normalization and smoothing

Our objective was to investigate whether acoustic measures and OQ during vowel intervals differed across registers. To facilitate comparison across speakers, f0, F1, F2, H1*-A3*, CPP, and OQ were converted to z-scores by speaker. Statistical analyses were performed on the z-scored values. However, for illustration purposes, we convert z-scores back to the original scales based on the grand mean and mean of standard deviations of all speakers (grand mean + z-score × mean standard deviation).

**Figure 2:**
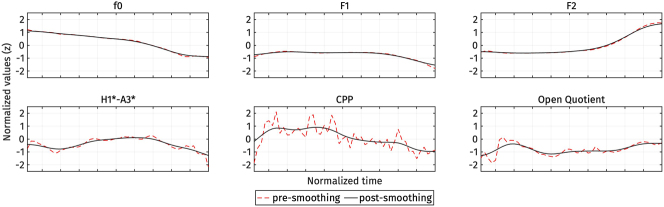
An example of trajectory smoothing for a high register token. Red dashed lines represent the time-normalized trajectories before smoothing, while black lines represent the time-normalized trajectories after smoothing.

We time-warped each trajectory to an equal length of 50 data points using MATLAB’s linear interpolation function interp1(). Since OQ data points were not sampled uniformly, the corresponding time steps for each OQ data point were also included as sample points in the linear interpolation process. The time-normalized trajectories were then further smoothed using MATLAB’s smoothdata() function, employing a Gaussian-weighted average over a window of 15 data points. This smoothing was applied to reduce ‘wiggliness’ caused by inaccurate or noisy measurements, as seen in Figure 2. These time normalization and smoothing steps are crucial for the subsequent functional principal components analysis (FPCA), as discussed in Gubian et al. (2015). For instance, CPP is inherently noisy, and the expected difference across registers pertains to the overall height of the trajectories rather than the noise variation in the trajectories. Smoothing helps prevent overfitting in the FPCA.

#### 2.1.6 Functional PCA

Acoustic and OQ trajectories were analyzed using functional principal components analysis (FPCA) (Ramsay and Silverman 2005) followed by linear mixed-effects modeling (Bates et al. 2015). We elected to use FPCA for three reasons. First, it allows us to model entire trajectories, rather than having to select arbitrary regions of the contour. Second, it produces easily interpretable parameterizations when compared to alternative methods such as GAMMs. Third, it permits the analysis of multidimensional dynamic trajectories, considering the changing shapes of multiple measurements simultaneously, such as vowel formants (F1 and F2) and voice quality measures (CPP, H1*-A3*, and OQ). For an introduction and overview of FPCA in a phonetics context, see Gubian et al. (2015).

FPCA provides a data-driven parameterization of one or more input trajectories. For the case of a single f0 trajectory, this parameterization can be expressed with the following equation:

(2) $$f0_{i}\left(t\right)\approx \mu_{f0}\left(t\right)+\overset{K}{\underset{k=1}{\sum }}s_{k,i}\cdot PCk_{f0}\left(t\right)$$

Here, *f*0_*i*_(*t*) represents the continuous function of f0 trajectories with *i* as the token index and *t* as the continuous normalized time variable, *μ*_*f*0_(*t*) is the mean f0 trajectory, *PCk*_*f*0_(*t*) are the *K* principal components (PCs) derived from the entire f0 trajectories (*k* = 1, …, *K*), for instance, *PC*1_*f*0_(*t*) is the first principle component derived from f0 trajectories, and *s*_*k*_ is the *score* that modulates *PCk* differently for each f0 trajectory. Each PC captures an independent mode of variation in the data, and each accompanying score controls the direction and magnitude of that variation. The significance of the scores can then be assessed using a linear mixed-effects regression in the usual manner. FPCA is similar to other functional methods of curve parameterization, such as the discrete cosine transform or growth curve analysis, but has the advantage that the PC curves are optimized for a particular dataset.

A major advantage of FPCA is that it can model multidimensional trajectories by combining tuples of functions like those in Eq. (2) (Happ and Greven 2018). Each dimension (trajectory) is parametrised by its own set of PCs, but the same set of PC *scores* is shared across dimensions. This means that each PC score jointly influences the shape of multiple dimensions, which is useful to model covariance between dimensions.

In this paper, we consider two multidimensional trajectories, one for vowel quality measures and one for voice quality measures. To analyse differences in vowel quality, we use as input trajectories for FPCA paired formant tracks, *F*1_*i*_(*t*) and *F*2_*i*_(*t*), defined as continuous functions representing F1 and F2, respectively. This can be expressed with the following equations:

(3a) $$F1_{i}\left(t\right)\approx \mu_{F1}\left(t\right)+\overset{K}{\underset{k=1}{\sum }}s_{k,i}\cdot PCk_{F1}\left(t\right)$$

(3b) $$F2_{i}\left(t\right)\approx \mu_{F2}\left(t\right)+\overset{K}{\underset{k=1}{\sum }}s_{k,i}\cdot PCk_{F2}\left(t\right)$$

The formulas for these paired functions are similar to the function for f0 trajectories, but both functions share the same *s*_*k*,*i*_ scores. In other words, a single *s*_*k*,*i*_ modulates both F1 and F2 for each token. In a similar fashion, we use FPCA to analyze voice quality measures by considering our three voice quality measures – CPP, H1*-A3*, and OQ – as three dimensions of the same phenomenon.

FPCA was applied to 2,004 tokens with voiceless stop onsets. Tokens with aspirated stop, preglottalized, and sonorant onsets were excluded from the analysis due to an imbalance in token numbers. The mean trajectories for each measurement across all onset categories, presented in Appendix B, suggest that registers are realized similarly across these categories, despite some differences in the magnitude of the effects.

#### 2.1.7 Statistical modeling of FPCA scores

Once all trajectories of all measurements were fitted with FPCA, we obtained the PC-scores *s*_1_ and *s*_2_, corresponding to the scores of *PC*1 and *PC*2, respectively. These PC-scores capture variations in the curves, irrespective of the register. We focused on the first two *PC*s because the PC scores of these higher components did not show any relationship with register distinctions. To investigate whether the scores differ across registers, the PC-scores *s*_1_ and *s*_2_ were modeled using linear mixed-effect regressions with the lmerTest package in R (Kuznetsova et al. 2017).

In these models, *s*_1_ and *s*_2_ for each set of measurements were included as dependent variables. The independent variable was register (high *vs.* low) for the models of PC-scores of f0 and voice quality. For the vowel quality models, vowel and its interaction with register were also included as independent variables. Subject was included as a random intercept and slope for register. The R model syntax is given in Eqs. (4a)–(4c).

(4a) $$s_{k\left(\text{f0}\right)}∼\text{register}+\left(\text{register}|\text{subject}\right)$$

(4b) $$s_{k\left(\text{Voice Quality}\right)}∼\text{register}+\left(\text{register}|\text{subject}\right)$$

(4c) $$s_{k\left(\text{Vowel Quality}\right)}∼\text{register}*\text{vowel}+\left(\text{register}|\text{subject}\right)$$

In Section 2.2, we present plots of the reconstructed trajectories based on model predictions with 95 % confidence intervals to provide an overview and demonstrate the significance of the estimates. These reconstructed trajectories were derived from the estimated marginal means of PC-scores, computed through post-hoc tests of the models showing significance using the emmeans package in R (Lenth 2022). In addition to model predictions, we reported both marginal and conditional *R* ^2^ values for each model, calculated using r.squaredGLMM from the MuMIn package (Bartoń 2024). Marginal *R* ^2^ represents the variance explained solely by the model’s fixed effects, while conditional *R* ^2^ represents the variance explained by the entire model, including both fixed and random effects.

The full model output is available in Appendix C.

### 2.2 Results

#### 2.2.1 Onset voicing

**Figure 3:**
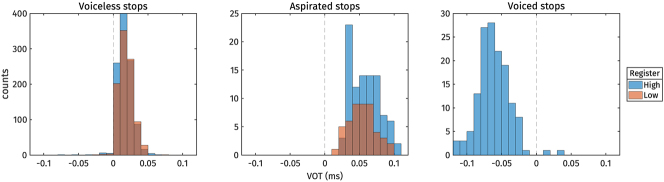
VOT distributions.

Before delving into the analyses of register effects on the vowels, we first checked whether registers were also associated with differences in onset voicing. Descriptive statistics for Voice Onset Time (VOT) are presented in Table 3 and illustrated as histograms in Figure 3. Within the categories of voiceless stops and aspirated stops, no observable effect of register on VOT was evident. Notably, in Figure 3, the VOT of aspirated stops in the high register appears slightly longer than in the low register. However, this difference is attributable to the presence of /k^h^/ in the high register and its absence in the low register. As shown in Table 2, /k^h^/ generally exhibits a longer VOT compared to /t^h^/. The expected difference in voicing across onset categories was observed, with voiceless stops exhibiting short positive voice lags, aspirated stops showing long positive voice lags, and voiced stops demonstrating voice leads. We identified just 19 tokens (out of 2,004) of voiceless stops exhibiting voice leads. It is worth noting that 13 of these tokens belonged to the high register, while the remaining 6 tokens belonged to the low register.

**Table 3:** VOT by voicing and register (in ms).

Onset	Register	Mean VOT	SD VOT
k	high	23	11
	low	25	8
t	high	11	7
	low	12	6
k^h^	high	67	19
t^h^	high	49	18
	low	54	19
ʔd	high	−61	23

**Figure 4:**
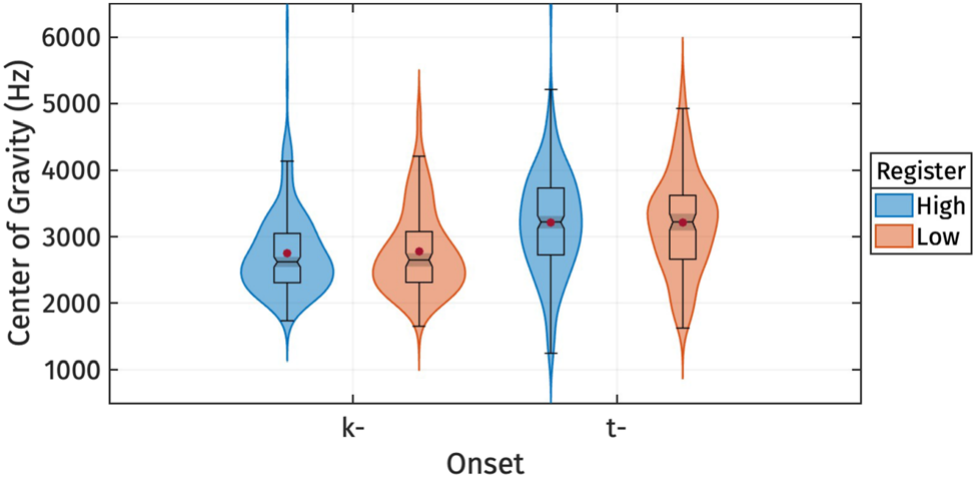
CoG of burst for different levels of register.

To investigate whether registers were associated with differences in onset voicing, we also examined the Center of Gravity (CoG) of burst, which has been linked to onset voicing by Chodroff and Wilson (2014). Descriptive statistics for CoG are presented in Table 4 and depicted as a boxplot in Figure 4. We focus on tokens with voiceless stops /t, k/ onset followed by the vowel /a/, which did not exhibit voice lead (total of 799 tokens). Within place of articulation, we did not observe any effect of register on CoG. The only difference in CoG observed was across place of articulation, with velar stop onset displaying a lower CoG than alveolar stop onset.

**Table 4:** CoG of burst by place of articulation and register (in ms).

Onset	Register	Mean CoG	SD CoG
k	high	2,754	628
	low	2,778	630
t	high	3,217	802
	low	3,211	749

In sum, the analysis of VOT and CoG distributions indicates that registers are not associated with acoustic differences in the onset voicing contrast.

#### 2.2.2 Vowels: f0

**Figure 5:**
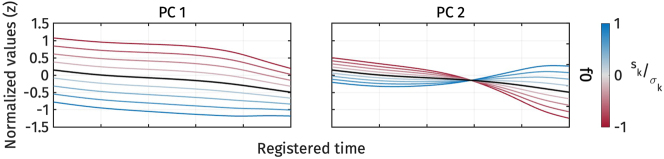
First two PCs of normalized f0 during target vowel interval pooled across speakers.

Figure 5 illustrates the shape variation of f0 captured by *PC*1 and *PC*2. Each panel represents the effect of each principal component. The dark solid line depicts the mean f0 trajectories, *μ*_*f*0_(*t*), with PC-scores *s*_*k*_ equal to zero. The color-coded curves represent the curves reconstructed from different *s*_*k*_ based on Eq. (2). Blue curves indicate the trajectories reconstructed from positive PC-scores, while red curves indicate those reconstructed from negative PC-scores. The *s*_*k*_ values used in Figure 5 are equally spaced, ranging between −1 (red) and +1 (blue) standard deviations (*σ*_*k*_).

The first two principal components explained 83.9 % and 13.9 % of the variance in all f0 trajectories, respectively. *PC*1 captures the height of f0 trajectories. A positive *s*_1_ corresponds to a lower f0 than the average f0 trajectory, whereas a negative *s*_1_ corresponds to a higher f0. On the other hand, *PC*2 captures the shape of the f0 contour, whether it is falling or rising. When *s*_2_ is positive, the f0 trajectory becomes rising, while a negative *s*_2_ reflects falling f0 contours.

**Figure 6:**
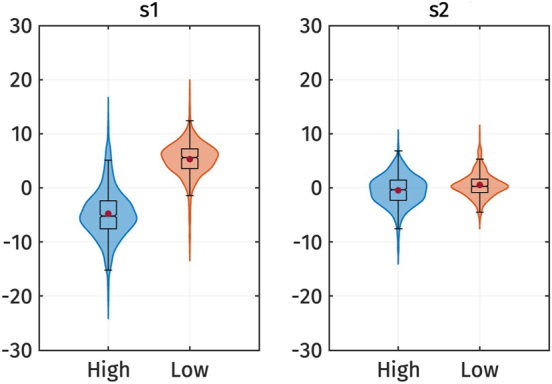
Violin plots of the *s*_1_ and *s*_2_ PC-scores of f0 by registers. Red dots represent the mean of each category.

Figure 6 illustrates the *s*_1_ and *s*_2_ scores for high and low registers. The figure shows that the high register exhibits lower *s*_1_ values compared to the low register, while the difference in *s*_2_ is not as pronounced.

This observation is also supported by the results of the linear mixed-effect regressions. Table 5 presents the predicted *s*_1_ and *s*_2_ scores based on the register. The effect of register on *s*_1_ is significant: *s*_1_ is higher for the low register, as indicated by the positive estimated effect size (*β*_1_), which is the difference in *s*_1_ between high and low register. This finding indicates that the high register has an overall higher f0 contour than the low register.

**Table 5:** Summary of the linear mixed effect regressions on the PC-score of f0. In all models, the baseline is high register. The *p*-values reported correspond to the *p*-values of the fixed effect (*β*_1_).

*s*_*k*_	*β*_0_	*β*_1_	*p*-values	Marginal *R* ^2^	Conditional *R* ^2^
*s*_1_	−4.80	10.09	<0.001	0.60	0.61
*s*_2_	−0.44	0.97	0.008	0.03	0.50

Although the effect of register on *s*_2_ is also significant, the marginal *R* ^2^ is very low, with the fixed effect of register explaining only 3 % of the variance in *s*_2_. In contrast, the conditional *R* ^2^ is much higher, indicating that the entire model, including both fixed and random effects, explains 50 % of the variance. The substantial difference between marginal and conditional *R* ^2^ suggests that the variation in *s*_2_ is primarily captured by the random effect, which is the speaker. In other words, *s*_2_ represents the variation across speakers rather than across registers. This suggests that the rising or falling contours of f0 are specific to individual speakers and not integral to the realization of registers.

**Figure 7:**
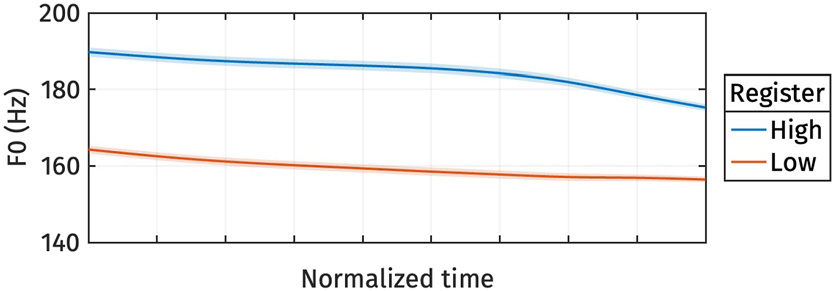
Reconstructed normalized f0 trajectories from the estimated marginal means of *s*_1_ for different registers.

The reconstructed f0 trajectories from the estimated marginal means of *s*_1_ are shown in Figure 7. The high register consistently exhibits a higher f0 than the low register throughout the entire trajectory.

#### 2.2.3 Vowels: voice quality measures

**Figure 8:**
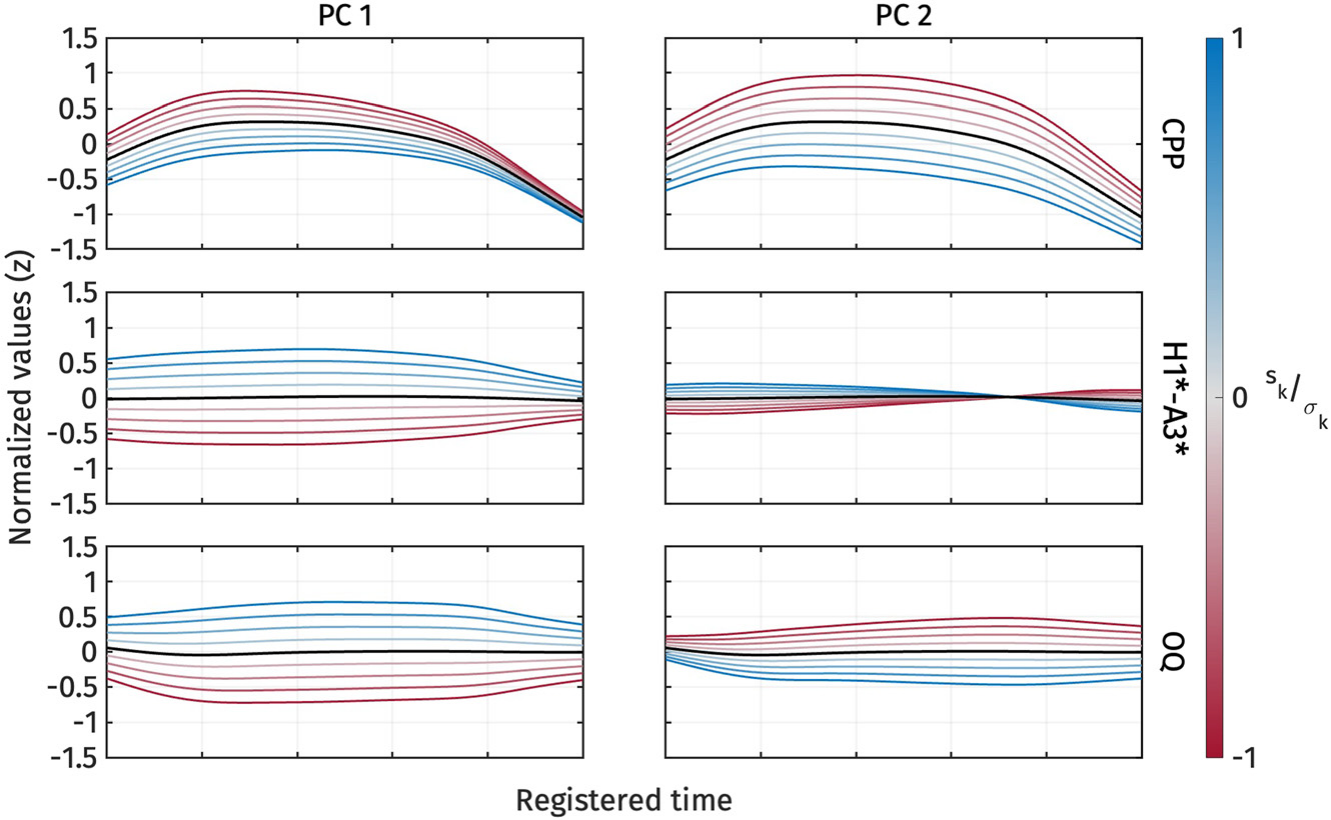
First two PCs of normalized CPP (top), normalized H1*-A3* (middle), and normalized OQ (bottom) during target vowel interval pooled across speakers.

Figure 8 illustrates the shape variation of the three voice quality measures, CPP, H1*-A3*, and OQ. The first two principal components explained 41.8 % and 25.7 % of the variance in all voice quality measure trajectories, respectively. For *PC*1 (Figure 8, left panel), an increase in *s*_1_ corresponds to a decrease in CPP and an increase in H1*-A3* and OQ. Conversely, a decrease in *s*_1_ corresponds to an increase in CPP and a decrease in H1*-A3* and OQ. In other words, *s*_1_ captures breathiness: high *s*_1_, indicating low CPP, high H1*-A3*, and high OQ, signifies breathy voice quality.

On the other hand, *PC*2 (Figure 8, right panel) represents variation in CPP and OQ. An increase in *s*_2_ indicates a decrease in CPP and OQ, and vice versa. We can interpret *PC*2 as capturing variation in how voice quality is realized across different measurements. For example, in some tokens, breathy voice quality might be indicated by low CPP, while OQ does not necessarily show a high value. *PC*2 does not seem to account for the variation in H1*-A3*, as changes in *s*_2_ do not result in large variations of H1*-A3*.

**Figure 9:**
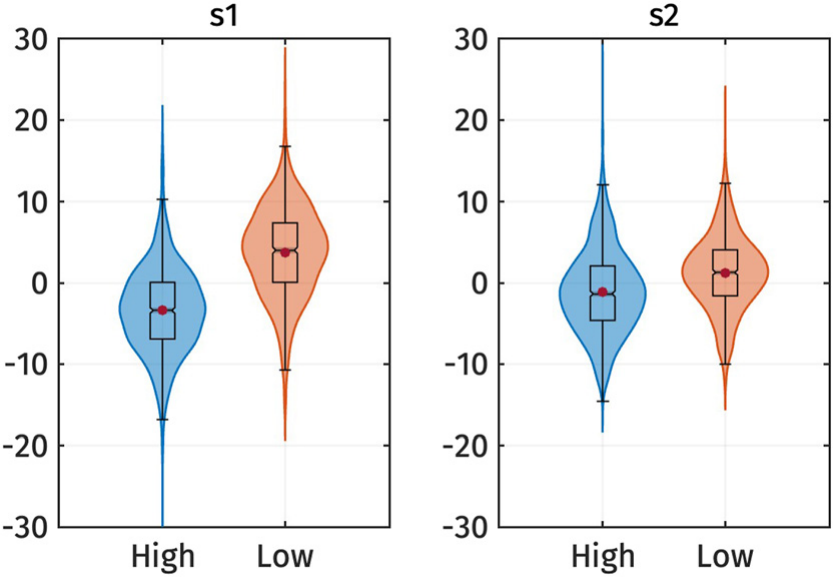
Violin plots of the *s*_1_ and *s*_2_ PC-scores of voice quality measures by registers. Red dots represent the mean of each category.

Figure 9 shows the *s*_1_ and *s*_2_ scores for high and low registers. The figure indicates that the high register has lower *s*_1_ values compared to the low register. Although *s*_2_ is also lower for the high register, the difference is less pronounced. This observation is supported by the results of the linear mixed-effect regressions.

Table 6 presents the predicted *s*_1_ and *s*_2_ scores based on register. The effect of register on *s*_1_ is significant: *s*_1_ is larger for the low register, as indicated by the positive estimated effect size (*β*_1_). This finding suggests that the low register has a breathier voice quality than the high register.

**Table 6:** Summary of the linear mixed effect regressions on the PC-score of voice quality measures. In all models, the baseline is high register. The *p*-values reported correspond to the *p*-values of the fixed effect (*β*_1_).

*s*_*k*_	*β*_0_	*β*_1_	*p*-values	Marginal *R* ^2^	Conditional *R* ^2^
*S*_1_	−3.39	7.12	<0.001	0.30	0.40
*s*_2_	−1.1	2.3	0.008	0.05	0.14

The effect of register on *s*_2_ is also significant, but the marginal *R* ^2^ is very low, with the fixed effect of register explaining only 5 % of the variance in *s*_2_. The conditional *R* ^2^ is slightly higher, indicating that the entire model, including both fixed and random effects, explains 14 % of the variance. This indicates that *s*_2_ represents variation across speakers rather than registers. Suggesting that voice quality is realized differently by different speakers. However, the effect is rather small, since the model still explains only 14 % of the variance.

**Figure 10:**
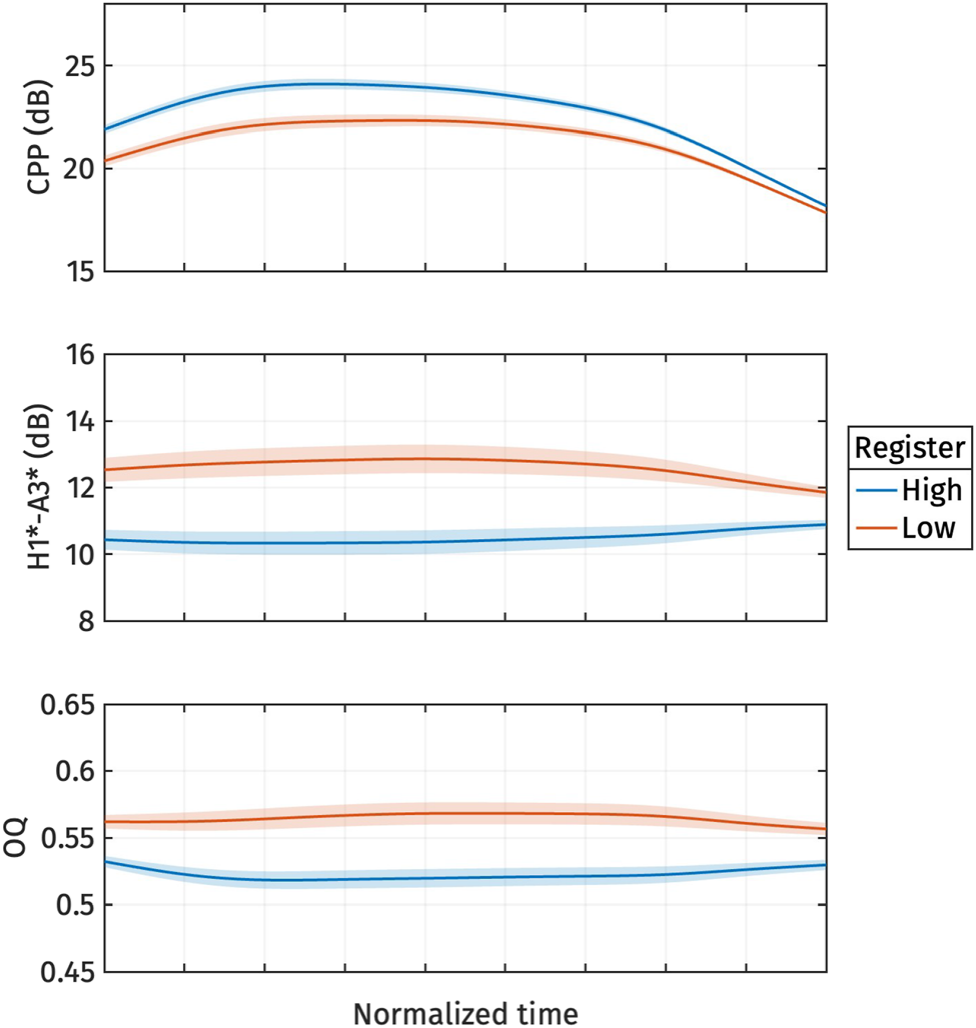
Reconstructed normalized CPP (top), normalized H1*-A3* (middle), and normalized OQ (bottom) trajectories from the estimated marginal means of *s*_1_ for different registers.

The reconstructed voice quality measures from the estimated marginal means of *s*_1_ are shown in Figure 10. High register tokens consistently exhibit higher CPP, and lower H1*-A3* and OQ than low register tokens. In other words, the high register shows more modal voice quality, while the low register shows breathy voice quality.

#### 2.2.4 Vowels: formant frequencies

**Figure 11:**
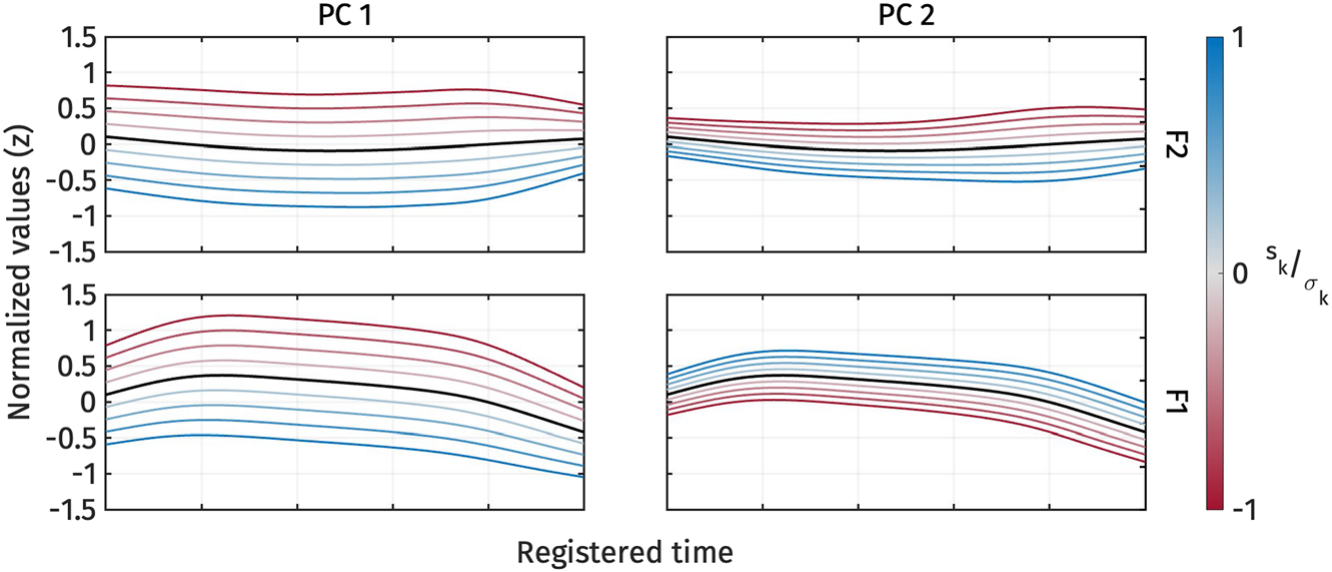
First two PCs of normalized F2 (top) and normalized F1 (bottom) during target vowel interval pooled across speakers.

Figure 11 illustrates the shape variation of vowel quality measures, F1 and F2, captured by *PC*1 and *PC*2. The first two principal components explained 71.3 % and 17.4 % of the variance in F1 and F2 trajectories, respectively. For *PC*1 (Figure 11, left panel), an increase or decrease in *s*_1_ corresponds to a decrease and increase in F1 and F2 together. An increase in *s*_1_ indicates the lowering of both F1 and F2 (blue trajectories). In other words, *s*_1_ captures vowel height and frontness: a high *s*_1_, indicating low F1 and low F2, signifies a higher and more back vowel.

On the other hand, for *PC*2 (Figure 11, right panel), the increase and decrease cause F1 and F2 to come closer together (blue trajectories) or shift further apart (red trajectories). Therefore, the modulation of *s*_2_ also captures vowel height and frontness again: a high *s*_2_, indicating high F1 and low F2, signifies a higher and more front vowel.

**Figure 12:**
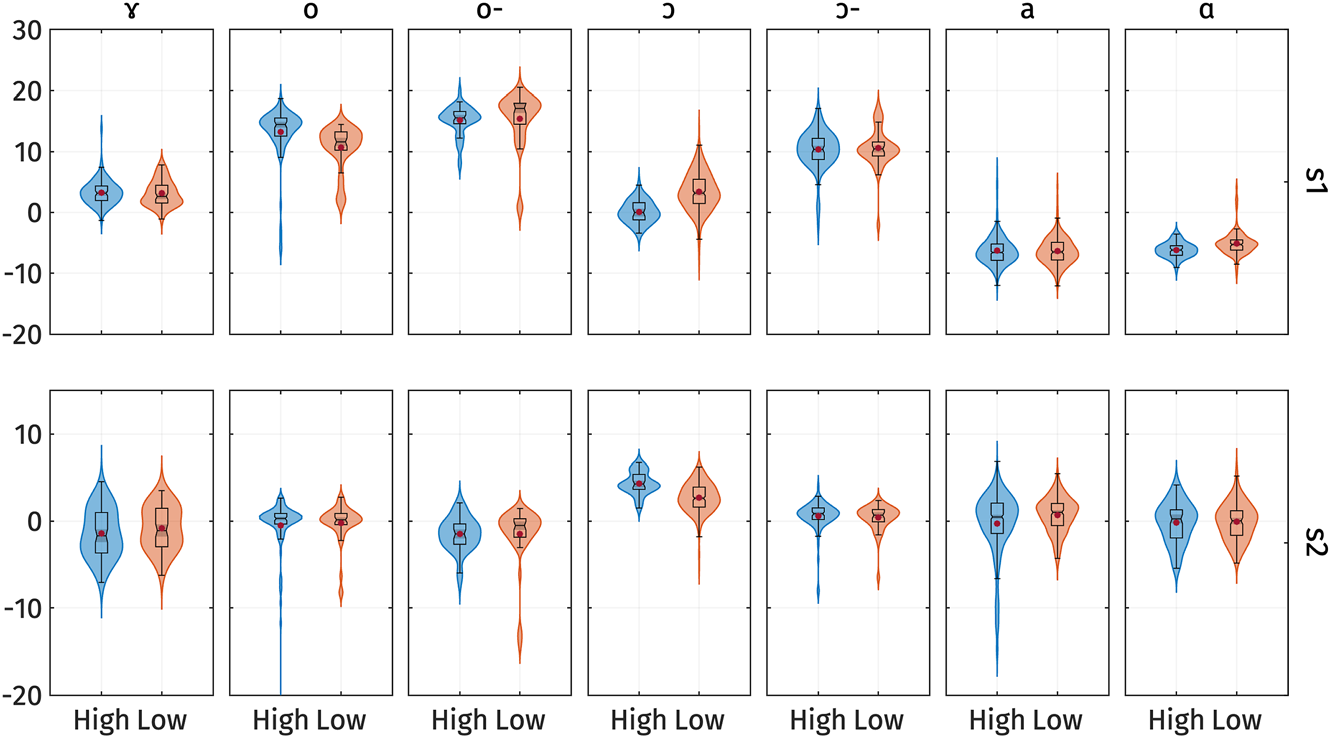
Violin plots of the *s*_1_ (top) and *s*_2_ PC-scores (bottom) of vowel quality measures by registers separated by vowels. Red dots represent the mean of each category. o- and ɔ- represents /o/ and /ɔ/ in open syllables respectively.

Figure 12 shows the *s*_1_ and *s*_2_ scores for high and low registers. The figure indicates that only a few vowels exhibit prominent differences in PC-scores. Furthermore, the vowels that do show differences do not consistently exhibit the same direction of the effect. This observation is supported by the results of the linear mixed-effect regressions.

The full model results are summarized in Appendix C. All models have the vowel /a/ with high register as the baseline. The model predicting *s*_1_ explains 88.1 % of the variance with only fixed effects and 88.4 % with both fixed and random effects. The effect of register on *s*_1_ is not significant (*t*(33.0) = −0.55, *p* = 0.59), suggesting that there is no difference in vowel formants across registers for the vowel /a/. We observed significant effects of the interaction between register and the vowels /o/ in closed syllables (*t*(1,888.3) = −4.91, *p* < 0.001), /ɔ/ in closed syllables (*t*(1,888.3) = 7.38, *p* < 0.001), and /ɑ/ (*t*(1,888.4) = 2.72, *p* = 0.007). This indicates that only the vowels /o/ in closed syllables, /ɔ/ in closed syllables, and /ɑ/ show differences in *s*_1_ across registers. However, the direction of the effect varies for different vowels. Specifically, /o/ in closed syllables shows a negative effect of register, indicating that the low register has lower *s*_1_, i.e., higher formant frequencies, which correspond to lower and more front vowels. In contrast, /ɑ/ and /ɔ/ in closed syllables show a positive effect of register, indicating that the low register has higher *s*_1_, i.e., lower formant frequencies, which correspond to higher and more back vowels.

On the other hand, the model predicting *s*_2_ explains 19.9 % of the variance with only fixed effects and 20.6 % with both fixed and random effects. The effect of register on *s*_2_ is significant (*t*(60.6) = 4.16, *p* < 0.001), suggesting that there is a difference in vowel formants across registers for the vowel /a/. We also observed significant interactions between register and the vowels /ɔ/ in closed syllables (*t*(1,890.3) = −5.63, *p* < 0.001) and also in open syllables (*t*(1,893.4) = −2.23, *p* = 0.026), and /ɑ/ (*t*(1,890.3) = −2.17, *p* = 0.03). To confirm whether the effect of register is significant for /ɔ/ and /ɑ/, we extracted the estimated marginal means using the emmeans package (Lenth 2022) in R. The marginal means of *s*_2_ with 95 % confidence intervals for each register are as follows: /ɔ/ in closed syllables High register: 4.4 [*CI*_95 %_ 3.6 5.1]; Low register: 2.7 [2.4 3.1]; /ɑ/ High register: −0.3 [−0.5 −0.04]; Low register: 0.7 [0.3 1.1]. The confidence intervals for high and low registers do not overlap for these vowels and the vowel /a/, while they do for all other vowels. This indicates that only /a/, /ɔ/, and /ɑ/ show significant differences in *s*_2_ across registers. However, the effect is rather small, since the model still explains only 19 % of the variance.

**Figure 13:**
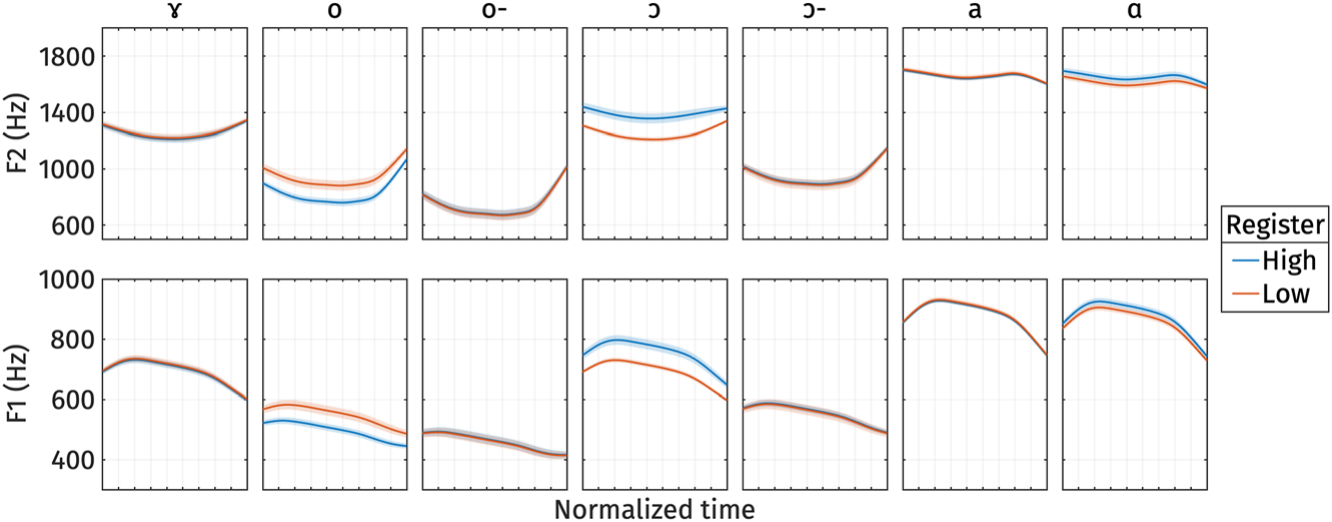
Reconstructed normalized F2 (top) and normalized F1 (bottom) trajectories from the estimated marginal means of *s*_1_ for different registers. o- and ɔ- represents /o/ and /ɔ/ in open syllables respectively.

The reconstructed formant frequency trajectories from the estimated marginal means of *s*_1_ are shown in Figure 13. Although *s*_2_ also shows differences for certain vowels, the effect size is small, and the model explains only a small proportion of variance (*R* ^2^), so we do not consider it further in our interpretation. As described in the analyses above, only three vowels exhibit differences in formant frequency across registers when considering *s*_1_. However, the direction of the effect is inconsistent among these vowels. Based on the typology of phonetic properties of register, only /ɔ/ in closed syllables and /ɑ/ show the expected direction of the effect (higher vowels in the low register). This suggests that vowel quality is likely not a distinguishing property of registers. The difference in vowel quality across register that we observed might be due to independent factors.

#### 2.2.5 Relation between f0 and voice quality measures

In the previous sections, we observed consistent differences in f0 and voice quality across registers. An interesting question that arises is whether f0 and voice quality are related or if they vary independently. In this section, we apply multidimensional FPCA to both f0 and voice quality measures to investigate whether variations in one correlate with variations in the other.

**Figure 14:**
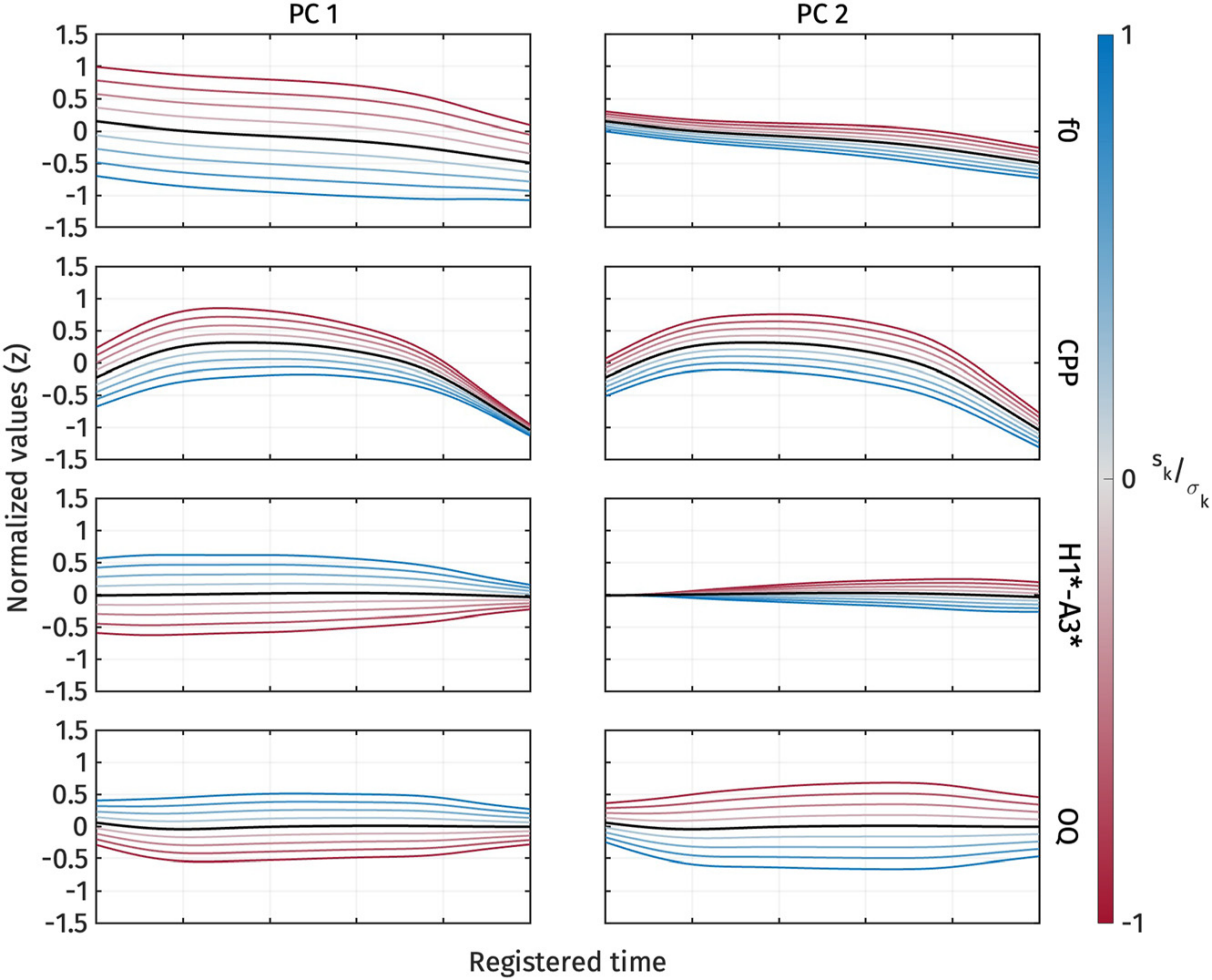
First two PCs of normalized f0, normalized CPP, normalized H1*-A3*, and normalized OQ during target vowel interval pooled across speakers.

Figure 14 illustrates the shape variation in f0 and voice quality measures as captured by *PC*1 and *PC*2. The first two principal components explained 47.3 % and 20.5 % of the variance in the combined f0 and voice quality trajectories, respectively. For *PC*1 (Figure 14, left panel), an increase in *s*_1_ corresponds to a decrease in f0 and CPP, along with an increase in H1*-A3* and OQ. Conversely, a decrease in *s*_1_ is associated with an increase in f0 and CPP, and a decrease in H1*-A3* and OQ. These directional variations align with the expected register contrasts: high register shows high f0, high CPP, low H1*-A3*, and low OQ, while low register shows the opposite pattern. Additionally, the standard deviations of *s*_1_ are comparable across all measurements, suggesting that variations in *s*_1_ induce changes in f0 and voice quality measures of similar magnitude.

In contrast, *PC*2 (Figure 14, right panel) primarily captures the simultaneous increase and decrease in CPP and OQ, similar to what was observed in the *PC*2 analysis of voice quality measures alone. An increase in *s*_2_ corresponds to a decrease in CPP and OQ, and vice versa. However, *PC*2 does not seem to account for variations in f0 and H1*-A3*, as changes in *s*_2_ do not lead to significant variations in these measures.

Table 7 presents the predicted *s*_1_ and *s*_2_ scores based on register. The effect of register on *s*_1_ is significant, with *s*_1_ being larger for the low register, as reflected by the positive estimated effect size (*β*_1_). This result suggests that the low register is associated with both a lower f0 and a breathier voice quality compared to the high register.

**Table 7:** Summary of the linear mixed effect regressions on the PC-score of f0 and voice quality measures. In all models, the baseline is high register. The *p*-values reported correspond to the *p*-values of the fixed effect (*β*_1_).

*s*_*k*_	*β*_0_	*β*_1_	*p*-values	Marginal *R* ^2^	Conditional *R* ^2^
*s*_1_	−5.92	12.44	<0.001	0.59	0.62
*s*_2_	−0.73	1.54	0.09	0.02	0.13

In contrast, the effect of register on *s*_2_ is not significant, and the marginal *R* ^2^ is quite low, indicating that the fixed effect of register accounts for only 2 % of the variance in *s*_2_. The conditional *R* ^2^ is slightly higher, suggesting that the full model, which includes both fixed and random effects, explains 13 % of the variance.

The results indicate a significant and comparable variation in both f0 and voice quality measures, as captured by *s*_1_, which supports the notion that these two dimensions are correlated. Changes in f0 are consistently associated with corresponding changes in voice quality across tokens. The significant effect of register on *s*_1_ further supports this finding, indicating that the relationship between f0 and voice quality is not only present but also meaningful in distinguishing between high and low registers. In other words, f0 and voice quality vary together in the production of register distinction.

#### 2.2.6 Inter-speaker variation in production

**Figure 15:**
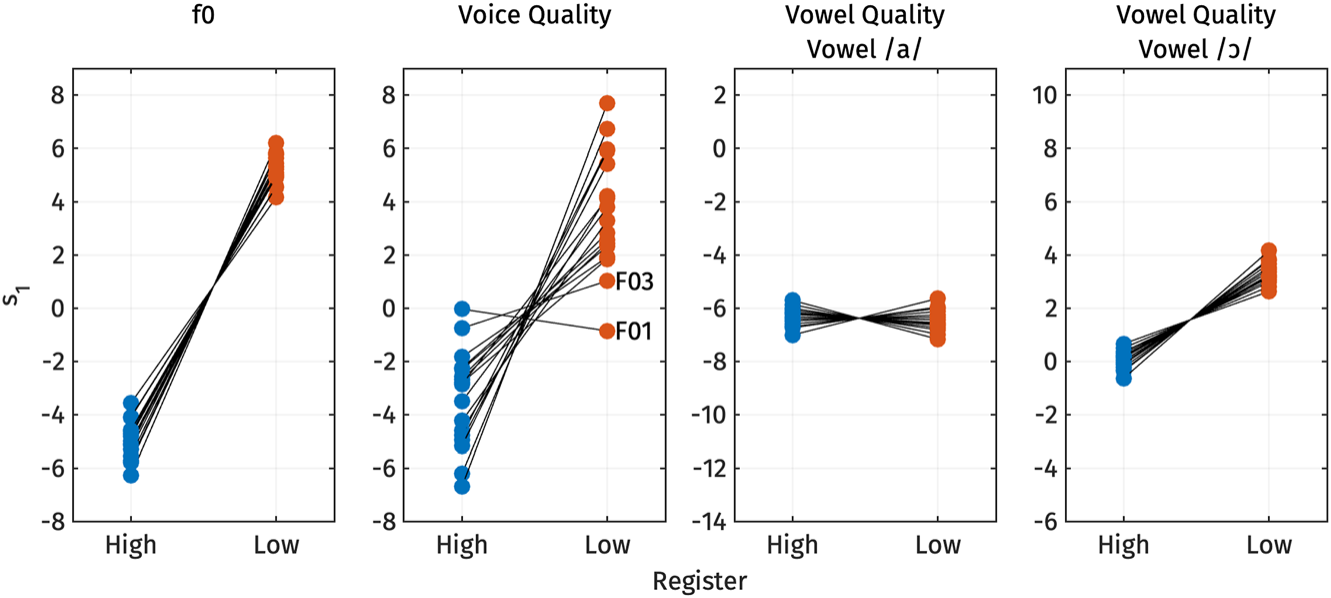
Predicted *s*_1_ values for f0, voice quality, and vowel quality across high and low registers for each speaker.

We also explored potential individual differences in the acoustic realization of register. Figure 15 shows the predicted *s*_1_ values for f0, voice quality, and vowel quality across high and low registers for each speaker from the linear mixed effect regression models discussed in Section 2.2.2, Section 2.2.3, and Section 2.2.4 above.

For f0, the variation in *s*_1_ across registers, which reflects overall f0 height, is pronounced and consistent across all speakers, with similar magnitudes. All speakers exhibit lower *s*_1_ values for the high register and higher *s*_1_ values for the low register, indicating that f0 is consistently higher for the high register and lower for the low register for all speakers.

In contrast, the difference in voice quality across speakers is less uniform. While most speakers display a clear difference in *s*_1_, with lower *s*_1_ values for the high register and higher *s*_1_ values for the low register – suggesting that the high register is more modal and the low register is breathier – there are exceptions. For example, speaker F01 does not follow the expected pattern, and speaker F03 exhibits only a slight difference between registers. This variation suggests that voice quality may not serve as a reliable acoustic correlate of register for these speakers.

Regarding vowel quality, Figure 15 shows the predicted *s*_1_ values for vowel /a/ (*n* = 761), which has the largest number of tokens, and for vowel /ɔ/ in closed syllables (*n* = 336), which was observed to be higher for the low register, as expected based on the typology outlined in Table 1. The figure indicates no systematic difference in vowel quality for vowel /a/. However, for vowel /ɔ/, all speakers show lower *s*_1_ values for the high register and higher *s*_1_ values for the low register, indicating that the vowel is consistently lower and more fronted for the high register, and higher and more backed for the low register, across all speakers.

One question regarding inter-speaker variation in register production that has been raised in previous literature is the relative weight of each register property. To address this, we also explore the relative contributions of f0, voice quality, and vowel quality to distinguish high and low registers. It is important to note that comparing the relative weight between f0 and voice quality may be problematic, given the strong correlation between these two properties, as discussed in Section 2.2.5. A more detailed discussion of this issue can be found in Section 4.3.

To compare the relative weight of each register property, we estimated the magnitude of the difference between high and low registers for each speaker using Cohen’s *d* (Cohen 1988). Cohen’s *d* was chosen because it normalizes effect sizes across different units of measurement, facilitating direct comparison. We first extracted the PC-score *s*_1_ for the f0, voice quality, and vowel quality trajectories. PC-score *s*_1_ was chosen over a raw data point from the trajectories because *s*_1_ represents the entire contour, arguably providing a more accurate description of the entire trajectory rather than focusing on an arbitrary part of the contour. Following the approach of Brunelle et al. (2020), we then calculated the vowel-weighted mean difference between the two registers for *s*_1_ of each trajectory, divided by the pooled mean standard deviation weighted by vowel and by register. While this measure is straightforward to compute, it is crucial to interpret the results cautiously, as potential correlations, particularly between f0 and voice quality, are not accounted for in this approach.

**Figure 16:**
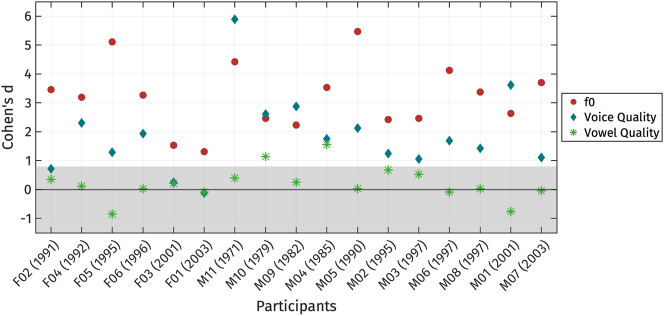
Individual variation in the use of acoustic properties distinguishing high and low registers, presented as Cohen’s *d* scores calculated from PC-score *s*_1_. Cohen’s *d* values have been multiplied by −1. Shaded area represents the area where Cohen’s *d* is less than 0.8. Participants are arranged based on their gender and year of birth.

Figure 16 displays the Cohen’s *d* scores for each production property, with all scores multiplied by −1 so that positive values represent the effect size in the expected direction of register differences. Scores below zero indicate that the direction of the effect for a given measurement is opposite to what is expected for register properties. Following Cohen (1988), scores lower than 0.8 are considered not meaningful.

The effect size of f0 is large for all speakers, indicating that f0 is one of the main properties for distinguishing register. Among the 17 speakers, 14 exhibit f0 as the most reliable acoustic correlate of register. However, three speakers (M01, M09, and M11) show the largest effect size for voice quality measures, with M10 also displaying a similar effect size between f0 and voice quality. Interestingly, M09, M10, and M11 – the speakers with large effect sizes for voice quality – are the oldest participants, whereas M01, who also exhibits a strong effect for voice quality, is among the youngest. This variation makes it difficult to determine whether age influences the production of voice quality. Several other speakers also show voice quality as a secondary property, as indicated by the second-largest effect sizes. On the other hand, some speakers, such as F01, F02, and F03, display relatively small effect sizes for voice quality measures, with Cohen’s *d* values below 0.8, suggesting that voice quality may not be a significant distinguishing feature of registers for these individuals. Notably, all male participants exhibit meaningful effect sizes for voice quality, whereas only three female participants show similar patterns. We did not observe a systematic trade-off observed between f0 and voice quality measures across speakers.

Vowel quality did not emerge as a robust correlate of register for most speakers, with none except M10 and M04 exhibiting Cohen’s *d* values greater than 0.8. M10 and M04 alone shows a relatively small difference in vowel quality between registers. No clear patterns related to gender or geographical origin were observed in the results.

### 2.3 Summary of the production results

Our findings show that fundamental frequency (f0) is a robust acoustic indicator of register for all speakers in our sample. This effect was pronounced, with notably large effect sizes (25–30 Hz), surpassing the reported f0 differences (<15 Hz) in previous studies of Mon register (Abramson et al. 2015; Thongkum 1987). We found limited evidence indicating that vowel quality is a distinguishing property of registers, contrary to a previous report (Behr 2018). Finally, while the low register consistently exhibited a breathier voice quality, indicated by lower CPP, higher H1*-A3*, and larger OQ than the high register, the effect sizes of voice quality measures were smaller than those observed by Abramson et al. (2015).

Regarding inter-speaker variation, our analysis revealed both consistent and inconsistent patterns across speakers. Differences in f0 between two registers were pronounced for all speakers. Additionally, voice quality was also identified as a property distinguishing the two registers, with three male speakers exhibiting it as the most acoustically prominent property, while three female speakers did not exhibit it as a property for registers. Furthermore, vowel quality did not emerge as a distinguishing cue across speakers, as evidenced by the small effect sizes observed for F1 and F2 in all speakers.

## 3 Perception experiment

### 3.1 Methods

#### 3.1.1 Participants, stimuli, and data collection

The perception experiment involved 29 listeners (10 females and 19 males). Among these participants, twelve had also taken part in the production experiment. The birth years of the participants ranged from 1971 to 2002 (21–52 years old at the time of the experiment). They were from various townships in Mon state: 10 participants were from Paung township, 7 from Thanbyuzayat township, 3 from Mudon township, 3 from Ye township, 2 from Kyaikmaraw township, 2 from Mawlamyine township, 1 from Chaungzon township, and 1 from Thaton township (see Figure 1). All participants were native speakers of Mon, and reported Mon to be their primary language. They also demonstrated fluency in Burmese and possessed basic proficiency in Thai.

Following the methodology of Brunelle et al. (2020), participants completed a forced-choice identification task, which involved listening to synthesized stimuli varying in fundamental frequency (f0), first formant (F1), and phonation type. They were then required to identify the stimuli by pressing one of two keyboard buttons corresponding to images displayed on a laptop screen. The stimuli comprised two sets. In one set, participants had to choose between /tɑk/ ‘building’ and /tɑ̤k/ ‘poor’ (henceforth /ɑ/-stimuli), while in the other set, the choices were /tɔ/ ‘shaft, rod, handle’ and /tɔ̤/ ‘close; cover, hide’ (henceforth /ɔ/-stimuli). Responses from 11 participants to the second set of stimuli (/tɔ/ *vs.* /tɔ̤/) were omitted from analysis due to ambiguity regarding the image corresponding to /tɔ/. Subsequently, responses from 29 participants were analyzed for /ɑ/-stimuli, while responses from 18 participants were analyzed for /ɔ/-stimuli.

Stimuli were synthesized using KlattGrid synthesis in Praat version 6.3.10. The script to synthesize the stimuli was based on Brunelle et al. (2020). As pitch, voice quality, and vowel quality have all been previously implicated in acoustic descriptions of the Mon register contrast, we manipulated three associated Klatt parameters: fundamental frequency (f0), open quotient (OQ), and the first formant frequency (F1). To establish the range for f0 and OQ, maximum and minimum values were derived from measurements obtained from target tokens produced in isolation by a male Mon speaker during a pilot study conducted in 2019. Since F1 did not covary with register in the speech of the model speaker, F1 ranges were based on the values reported in Abramson et al. (2015) and Behr (2018). These parameters were then combined to create all possible combinations of acoustic properties. The total number of stimuli was 45 tokens per word pair. The three parameters were manipulated as follows:

– **f0**: Pitch targets were set at three points within the vowel intervals to replicate the falling pitch pattern observed in words in isolation: the onset of the vowel (f0 onset), 50 ms into the vowel (f0 at steady state), and 90 ms before the end of the vowel (f0 pre-offset). Five sets of pitch targets were defined to generate five distinct pitch contours, with each set consisting of three values representing f0 onset, f0 at steady state, and f0 pre-offset, respectively: (i) 200, 170, 130 Hz; (ii) 180, 160, 125 Hz; (iii) 160, 150, 120 Hz; (iv) 140, 140, 115 Hz; and (v) 120, 130, 110 Hz. A fixed target of 100 Hz was set at the end of the vowel.
– **Open Quotient**: OQ was set to be stable throughout the vowel intervals, with three possible values: 0.3, 0.4, 0.5.
– **F1**: The two vowel stimuli were configured with distinct F1 targets. For the /ɑ/-stimuli, three possible values were set during the steady state period, from 20 ms into the vowel to 90 ms before the end of the vowel: 900, 800, and 700 Hz. Fixed targets of 600 Hz and 350 Hz were set at the onset and end of the vowel, respectively, to model the F1 transition from consonant onset and from coda. For the /ɔ/-stimuli, as native speakers tend to exhibit a slight off-glide in the vowel (see Figure 26 in Appendix B), F1 targets were adjusted differently at 20 ms into the vowel and 90 ms before the end of the vowel to replicate F1 contours. Three sets of potential values were established: (i) 800, 600 Hz, (ii) 650, 525 Hz, and (iii) 500, 425 Hz. A fixed target of 600 Hz was set at the beginning of the vowel to represent the F1 transition from consonant onset.

**Figure 17:**
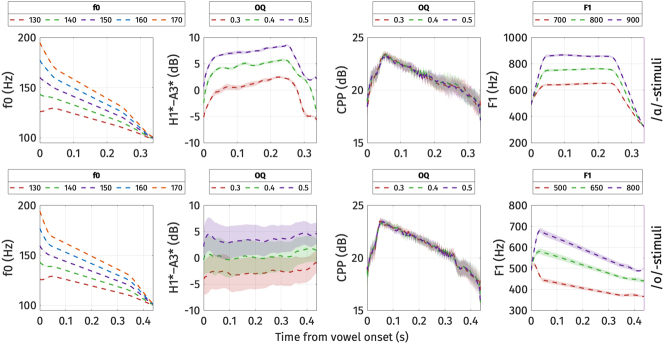
Means of the key acoustic parameters of the stimuli in the identification experiment. Top panel: /ɑ/-stimuli. Bottom panel: /ɔ/-stimuli. The ribbons correspond to one standard error above and below the mean.

The resulting stimuli were perceived as natural by the participants, with none reporting awareness that the stimuli had not been recorded from real speakers. Anecdotally, some participants reported that certain tokens sounded as if they were produced by a non-native speaker of Mon. Figure 17 illustrates the acoustic measurements of the resulting stimuli extracted using PraatSauce. Note that due to interactions among the parameters, the acoustic properties of the stimuli were not consistently precisely on target. In particular, the manipulation of OQ did not influence the CPP of the stimuli. However, given that the manipulation produced H1*-A3* values that were in line with the production data, combined with the fact that acoustic differences in H1*-A3* were much greater than those of CPP in the productions of the pilot speaker as well as in the production experiment, we judged the manipulations adequate.

The experiment was conducted using OpenSesame (Mathôt et al. 2012). Participants received instructions through visual aids and verbal explanations provided by a native Mon speaker. Before the testing phase, participants first completed a training phase using the two tokens with the most extreme parameter values, representing the expected natural production of the two registers (**high register**: highest f0, lowest OQ, highest F1; **low register**: lowest f0, highest OQ, lowest F1). Each stimulus was repeated three times. During training, participants listened to each stimulus while the corresponding image was highlighted as the audio played. Next, participants completed a first practice phase using the same two tokens as in the training phase, where they were asked to identify the stimuli by pressing the appropriate response buttons (5 repetitions per stimulus). During this phase, eight participants (F08, F09, F12, M03, M06, M09, M15, M16) had accuracy rates below 60 %. Finally, in a second practice phase, participants were presented with 10 randomly selected stimuli. These 10 random stimuli were drawn from the same pool as those used in the testing phase. Participants were encouraged to take short breaks between blocks.

In the testing phase, stimuli were presented in six blocks, with three blocks for each word pair alternating between /ɑ/- and /ɔ/-stimuli. Each block contained all 45 randomized stimuli for the relevant word pair, resulting in a total of 240 tokens per listener. The entire experiment took 15–20 minutes per participant. Responses with reaction times exceeding 2 seconds were not recorded.

#### 3.1.2 Statistical analyses

Responses were analyzed using mixed-effects logistic regressions implemented in the lmerTest package (Kuznetsova et al. 2017) in R. Separate models were fitted for each word pair. The dependent variable was participant response, indicating whether they chose the high or low register stimulus. Fixed effects included the f0, OQ, and F1 values of the synthesized stimuli, which were centered and treated as discrete numerical variables. For example, the five levels of f0 were coded as [−2, −1, 0, 1, 2], ordered from lowest to highest f0 of the stimuli. Two-way interactions between the fixed-effect variables were also included in the maximal models. Subjects were included as random intercepts with random slopes for all non-interaction fixed effects. The maximal model can be expressed with R syntax as follows:

(5) $$\begin{array}{ll} \text{Responses} & ∼\text{f0}+\text{OQ}+\text{F}1+\left(\text{f0 }*\text{OQ}\right)+\left(\text{f0 }*\text{F}1\right)+\left(\text{OQ }*\text{F}1\right)\\ & \;+\left(\text{f0}|\text{Subject}\right)+\left(\text{OQ}|\text{Subject}\right)+\left(\text{F}1|\text{Subject}\right) \end{array}$$

We began by fitting the maximal model, which included all possible interactions and fixed effects. The model was then simplified through a step-wise process, sequentially removing the interaction or fixed effect with the lowest *F*-value. Interactions were removed first, followed by fixed effects. The final, simplified models were those that did not result in a significant increase in the Akaike Information Criterion (AIC) score when compared to the more complex models. Throughout the process, the random effect structure was kept unchanged.

### 3.2 Results

#### 3.2.1 Identification experiment

**Figure 18:**
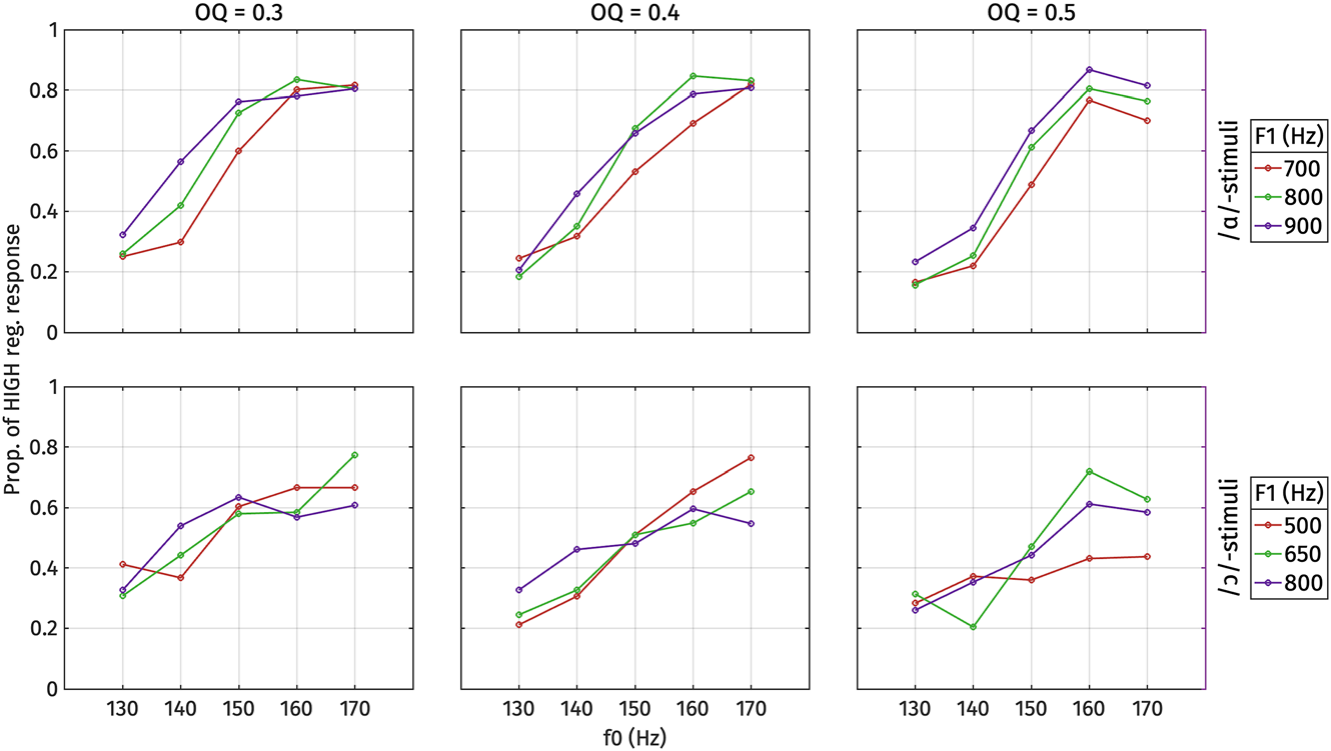
Proportion of high register responses for each type of stimulus, by F1, f0, and open quotient (OQ), averaged over all listeners. Top: /ɑ/-stimuli. Bottom: /ɔ/-stimuli.

**Figure 19:**
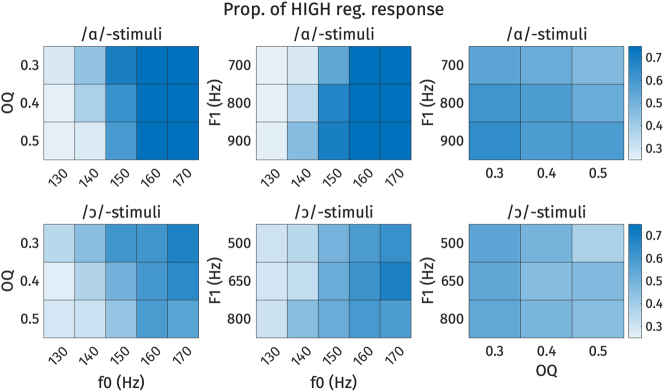
Heat plots of high register responses across each combination of f0, F1, and OQ. Each cell represents the proportion of high register responses for each stimulus. Darker shades represent higher proportion of high register responses. Top: /ɑ/-stimuli, bottom: /ɔ/-stimuli. Left: OQ by f0, middle: F1 by f0, right: F1 by OQ.

The proportion of high register responses obtained from listeners is illustrated in Figures 18 and 19. Summaries of the final logistic regression models are provided in Table 8 for /ɑ/-stimuli and Table 9 for /ɔ/-stimuli.

**Table 8:** Table of estimates of the final logistic regression model for /ɑ/-stimuli.

	Estimate	Std. error	*z* value	Pr(>|*z*|)
(Intercept)	0.56	0.17	3.36	<0.001
f0	1.12	0.16	7.28	<0.001
OQ	−0.32	0.10	−3.45	<0.001
F1	0.35	0.11	3.21	0.001

**Table 9:** Table of estimates of the final logistic regression model for /ɔ/-stimuli.

	Estimate	Std. error	*z* value	Pr(>|*z*|)
(Intercept)	−0.02	0.16	−0.14	0.89
f0	0.54	0.18	3.05	0.002
OQ	−0.28	0.07	−4.29	<0.001

For /ɑ/-stimuli, the final model includes fixed effects of f0, OQ, and F1, but no interaction terms. Among these factors, f0 demonstrates the largest effect size, indicating its strongest weight on the perceptual responses. The effect sizes of OQ and F1 are comparable, suggesting equally robust effects. Specifically, positive correlations are observed between f0 and F1 and high register responses, indicating that higher f0 and F1 values correspond to a higher proportion of high register responses, as expected based on the typology and the production results. Conversely, OQ exhibits a negative correlation with high register responses, implying that higher OQ values (indicating breathier phonation) correspond to a lower proportion of high register responses.

For /ɔ/-stimuli, the final model includes fixed effects of f0 and OQ only, but not an effect of F1 or any interaction terms. Similar to /ɑ/-stimuli, f0 demonstrates the largest effect size and is positively correlated with high register responses. Similarly, OQ exhibits a negative correlation with high register responses, consistent with the patterns observed in /ɑ/-stimuli.The lack of a significant F1 effect in the perception of /ɔ/-stimuli is consistent with the observed production behavior of /ɔ/ in open syllables.

The analysis of conflicting cues in Figures 18 and 19 demonstrates how listeners navigate ambiguity when acoustic cues suggest different register categories. For the /ɑ/-stimuli, when f0 is 170 Hz (indicating high register) or 130 Hz (indicating low register), listeners tended to respond accordingly, regardless of whether the F1 or OQ values conflict. When f0 is ambiguous, however, small effects of both F1 and OQ can be observed. For the /ɔ/-stimuli, responses to stimuli with ambiguous f0 values (140–150 Hz) tended to be around chance, although here again the likelihood of a high response decreased when OQ was indicative of low register.

These findings suggest that when faced with conflicting cues, listeners tend to make preferential use of the f0 cue. However, there is also evidence that they attend to voice and vowel quality cues, especially if f0 is indeterminate.

#### 3.2.2 Inter-speaker variation in perception

In line with the production experiments, we also investigated potential individual differences in perception. Initially, logistic regressions were fitted for each listener, with fixed effects of f0, OQ, and F1 on the two sets of perception stimuli. Due to the limited number of tokens tested with each participant, no interactions or random effects were included. We interpret the coefficients of the logistic regression as the perceptual weights of each perception parameter, following previous works (e.g., Brunelle et al. 2020; Schertz et al. 2015).

**Figure 20:**
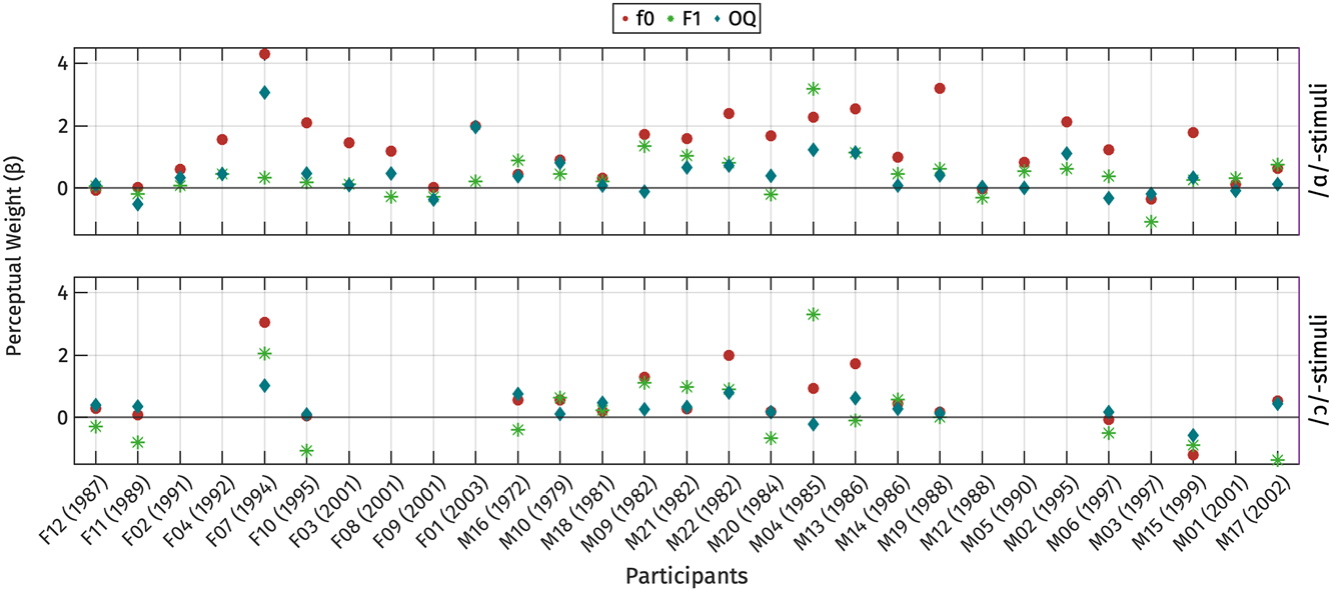
Individual variation in the use of perceptual properties to distinguish between high and low registers, presented through the coefficients from logistic regression models. Coefficients associated with OQ have been multiplied by −1 for clarity. Participants are arranged based on their gender and year of birth.

The coefficients for each participant are illustrated in Figure 20 for each perception parameter. Coefficients for OQ have been multiplied by −1, so that positive values indicate effect sizes in the expected direction, and values below zero suggest that the direction of the effect is unexpected. A perceptual weight greater than zero indicates that the participant used the parameter to distinguish between the two registers.

As shown in Figure 20, some listeners were unable to differentiate between the two register categories using the manipulated parameters (F09, F11, F12, M01, M06, M18, M19). This is evident from the clustering of perceptual weights around zero for all parameters. This could mean that their perceptual strategies for distinguishing registers may not rely on the manipulated parameters, but could also indicate that our manipulation was in some respects unsuccessful. It is worth noting that, among these seven participants, only three participants (F09, F12, M06) had accuracy rates below 60 % in the practice phase.

For the majority of participants (F03, F04, F08, M02, M03, M05, M09, M12, M13, M15, M22), f0 was the cue with the highest perceptual weight. Notably, one listener (M04) consistently relied on F1 differences across both sets of stimuli to distinguish the two registers.

Voice quality, at least as implemented in terms of OQ differences, was not found to be the most strongly weighted parameter for any of our listeners. While both F1 and OQ typically have non-zero weights, the precise weighting varies considerably across speakers. Some participants exhibited a stronger perceptual weight for F1 over OQ, while others demonstrated the opposite pattern. We did not find any correlation between perceptual weights and participants’ gender or year of birth.

Heat plots for each listener can be found in Appendix D.

### 3.3 Summary of the perception results

Our findings reveal variability in the perception of Mon register. Generally, listeners relied on fundamental frequency (f0) and voice quality to distinguish between registers, while vowel height (F1) influenced the perception of register only for /ɑ/-stimuli, and then primarily when f0 was ambiguous. Consistent with the production results, f0 seems more heavily weighted as a cue to register compared to voice and vowel quality, and listeners tended to prioritize f0 over other acoustic cues when cues were in conflict.

Regarding listener-specific variation, aside from participants who effectively failed to distinguish between high and low registers in our experiment, almost all participants primarily relied on f0 as the most salient cue. Listeners also exhibited variability in the weight assigned to other acoustic cues for distinguishing registers. Specifically, while some listeners used formant frequency (F1) and voice quality (as represented by differences in OQ) to some extent, only one listener relied primarily on F1, and none relied on voice quality as the most salient cue.

### 3.4 Relation between production and perception

Finally, we investigated the relationship between the production and perception of register for the twelve Mon participants who completed both production and perception tasks. As only four participants from this group also completed the perception experiment with /ɔ/-stimuli, we focus here exclusively on responses to the /ɑ/-stimuli.

Our exploratory analysis took the form of a simple correlation between production and perception weights, a la Shultz et al. (2012). For perceptual weights, we used the coefficients obtained from logistic regressions, as detailed in Section 3.2.2. The production weights were determined using Cohen’s *d* values. These were calculated in a manner similar to that in Section 2.2.6, but specifically focused on the subset of tokens from the vowel /ɑ/.

**Figure 21:**
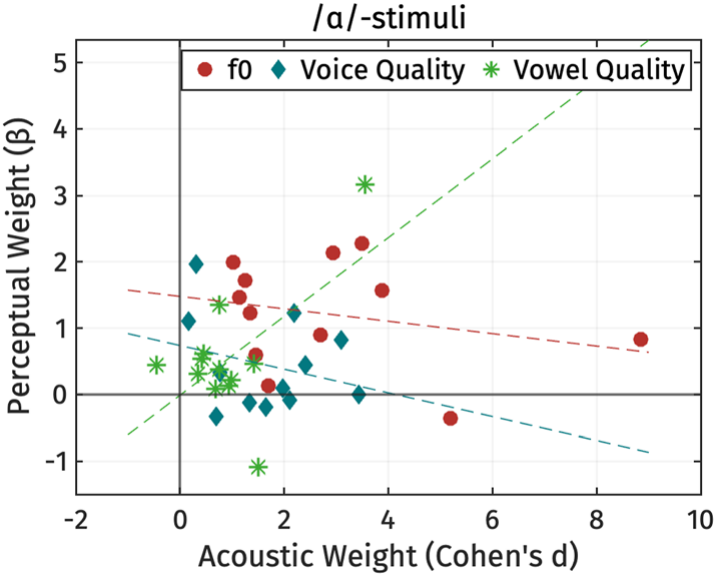
The correlation between perceptual weights (*β*) for /ɑ/-stimuli and acoustic weights (Cohen’s *d*) in individual participants. Voice quality represents the PC-score *s*_1_ of voice quality and OQ of the perception stimuli. Cohen’s *d* of all measures and coefficients of perception OQ have been multiplied by −1. Dashed lines represent least-squared line between production and perception weights.

**Figure 22:**
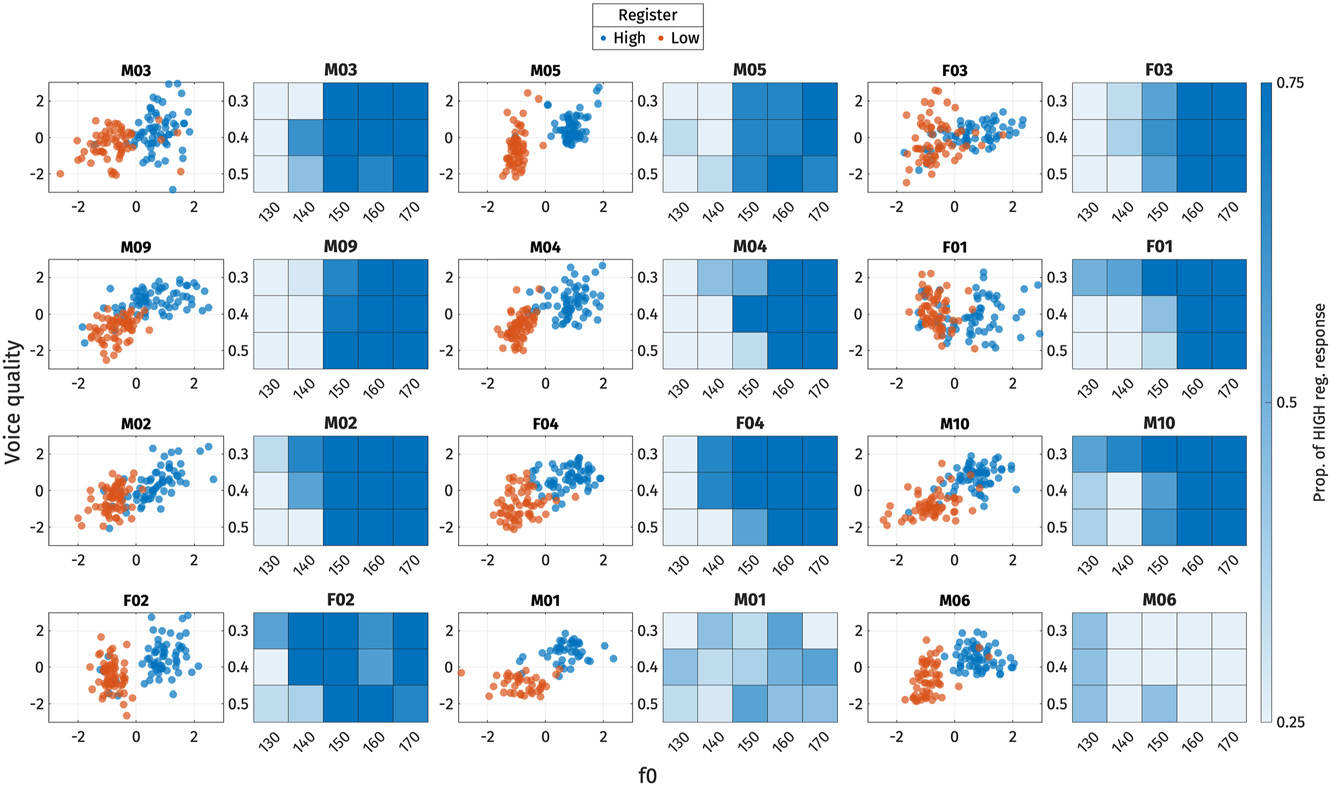
Relationship between the production and perception shown in scatter plots of PC-scores *s*_1_ (multiplied by −1 to match with the perception heat plots) and heat plots of high register responses on /ɑ/-stimuli. Voice quality represents the *s*_1_ of voice quality measures in the production and OQ of the perception stimuli.

The outcomes of this analysis are shown in Figure 21. Here, the weight of each relevant acoustic property (Cohen’s *d*) is plotted on the *x*-axis, while the weight of the corresponding perceptual property (*β*) is plotted on the *y*-axis. Each participant is depicted by a data point in the scatter plots. Individual heat plots of their responses in the perception experiment, illustrating the relationship between f0 and voice quality cues, are provided alongside the individual production graphs showing the same relationship in Figure 22.

As shown in Figures 21 and 22, participants who exhibit f0 as a cue to register in production also tend to rely on f0 in perception, as evidenced by their positive values for both acoustic and perception weights. However, there are exceptions, such as participants M01 and M06, who exhibit positive weights for f0 in production but not in perception. This discrepancy may be due to the fact that these two participants did not systematically distinguish between high and low registers in the perception experiment (as shown in Figures 20 and 22).

Regarding voice quality, some participants, like F04 and M10, demonstrate positive weights for both production and perception. However, the majority either use voice quality cues exclusively in production or in perception. Similarly, for vowel quality, a few participants display weights close to zero in both production and perception, or show a positive weight in one domain and a negative weight in the other. The exception is M04, who exhibits a salient positive weight for vowel quality in both production and perception. Notably, while the majority of speakers exhibit a mismatch between production and perception weights for vowel quality and voice quality cues, only a small number show such a discrepancy for f0.

Globally, we did not observe any significant correlation between the acoustic and perception weights for any phonetic properties, based on Pearson’s *r* correlations. This suggests that the weight of participants’ perceptual cues cannot be predicted from the weight of the corresponding acoustic cues.

## 4 General discussion

### 4.1 Production and perception of Mon registers

Similar to previous studies on Mon registers (Abramson et al. 2015; Behr 2013, 2018; Lee 1983; Thongkum 1987, 1990), we observed a robust register contrast in the speech of our Burma Mon speakers, with fundamental frequency (f0) and voice quality as the main distinguishing properties. Fundamental frequency (f0) was consistently an important property of register in the productions of all speakers, while voice quality differences were absent for some speakers. The results of our perception experiment were similar: all listeners made clear use of f0 to distinguish between registers, while the use of voice and vowel quality was primarily restricted to stimuli with ambiguous f0 values. These findings enable us to answer our research questions as follows:

1) What is the acoustic realization of register in Mon? To what extent do the acoustic correlates of register covary? Is there any individual variation in the production of register?

The Mon register contrast can be characterized by differences in f0 and voice quality, as measured by H1*-A3*, CPP, and EGG open quotient. Vowel quality does not appear to play a significant or systematic role in distinguishing registers in this variety. Our FPCA analysis showed that f0 and acoustic measures of voice quality tend to covary.

In terms of individual variation, f0 differences distinguished high from low register tokens in the speech of all speakers. Most speakers also produced the register contrast with significant voice quality differences, but only three speakers had voice quality as the most robust production property. Even among these three, f0 remained significant, with an effect size comparable to that of voice quality.

2) What perceptual cues do Mon speakers use to distinguish high and low register? Is there any individual variation in perception?

We observed that f0 and voice quality are both used as perceptual cues to register, with f0 typically having the largest effect size. F1 was found to be a distinguishing cue for only vowel /ɑ/. It is worth noting that /ɑ/ shows difference in vowel quality in the production of registers with expected direction of the effect. Therefore, vowel quality might be a distinguishing cue to registers for some vowels but not others. Furthermore, the analysis of conflicting cues suggests that listener tended to rely on f0 other cues took on values suggestive of different register categories.

Regarding listener-specific variations, most listeners who were able to distinguish the two registers in our experiment relied on f0 as the most prominent cue. However, one listener primarily used F1 to distinguish the registers. None of the listeners relied primarily on voice quality. However, we cannot rule out the possibility that our synthesized stimuli simply failed to reproduce the spectral differences accurately enough for some listeners.

3) Is there any correlation between the prominence of acoustic properties in production and their salience in perception?

As only a small subset of our participants completed both tasks, our answer to this research question must be interpreted with caution. Participants who produced registers with noticeable f0 differences tended to rely on f0 for perception as well. However, for the majority of participants, voice quality was typically either prominent in production or salient in perception, but not both. One participant appeared to rely on vowel quality in both production and perception. Although most speakers showed inconsistency between production and perception weights or the weights close to zero for vowel quality and voice quality, few participants exhibited this inconsistency for f0. We did not observe any significant correlation between production and perception weights globally, which is likely expected since Mon speakers often perceive different speech characteristics within their community, not necessarily reflecting their own production patterns.

### 4.2 Differences from previous acoustic studies

Our findings highlight the importance of f0 as a cue to registers in Burma Mon, both in production and perception, aligning with previous acoustic studies of primarily Thai Mon varieties (Behr 2013; Lee 1983; Thongkum 1987, 1990). However, our findings differ from those of Abramson et al. (2015), who reported only a small acoustic difference in the production of f0 across registers and suggested that f0 is an automatic consequence of phonation type rather than a distinct cue to register. This raises a question: what accounts for the discrepancy between our findings and those of Abramson et al. (2015)?

One possible explanation for the discrepancy lies in dialectal differences. The studies by Abramson et al. (2015) and Thongkum (1987, 1990) focused on Thai Mon speakers from Nakornchum, Thailand, who are bilingual in Thai. In contrast, our data was collected exclusively from Burma Mon speakers. This geographical and linguistic variation might subsume regional differences in the prominence of various cues for the register distinction. The influence of dominant languages (Thai *versus* Burmese) may also contribute to these differences. However, this seems less likely since both Thai and Burmese are tonal languages that use f0 as a distinguishing cue for tones. Unfortunately, finding monolingual Mon speakers is virtually impossible, and all research on Mon, including ours, has necessarily involved multilingual participants.

Another factor to consider is the possibility of a shift in cue weighting due to sound change. In other words, what was once a small f0 difference, initially a by-product of phonation type, may have been phonologized into a distinctive cue for register. However, we note that earlier studies, such as Thongkum (1987, 1990), reported large f0 differences between the two registers in data collected from Mon speakers in the same community as Abramson et al. (2015).

### 4.3 Relationship between pitch and voice quality in the production of register

Research on register contrasts, including our own work detailed in Sections 2.2.6 and 3.2.2, often focuses on identifying the relative weight of acoustic cues that distinguish registers. Studies have typically debated whether f0, voice quality, vowel quality, or other acoustic properties serve as the primary or secondary cues for distinguishing registers in a particular language or variety. However, this approach may not fully capture the complexity of register, especially in production.

In Section 2.2.5, we established a statistical correlation between and f0 and voice quality measures, as variation in f0 was found to correspond with variation in CPP, H1*-A3*, and EGG-derived open quotient. This finding underscores the importance of taking seriously the notion of register as a multivariate acoustic property arising from synergistic articulatory contingencies. Indeed, covariance between multiple acoustic correlates of register is precisely what would be predicted if the articulatory target of high and low registers is a modal or lax vocal tract configuration, respectively. For example, in the case of breathy voice, there is a strong relationship between breathiness and low pitch due to both the reduced frequency of vocal fold vibration and the relative separation and looseness of the vocal folds (Esling et al. 2019; Laver 1980). During the production of breathy voice, the posterior half of the glottis typically remains open, while the anterior half does not. This configuration leads to the vibration of only the anterior half of the vocal folds, which contributes to a lower pitch, as does the overall slackness of the vocal folds.

**Figure 23:**
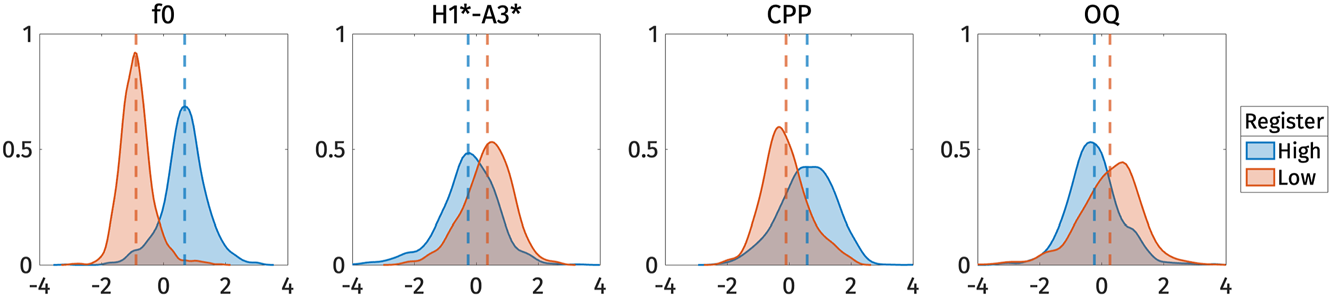
KS density plots showing distributions of f0, H1*-A3*, CPP, and OQ for high and low registers. Vertical lines represent the mean value of the measurements of the two registers.

From a purely acoustic perspective, differences in f0 are greater than differences in voice quality measures across registers, particularly when considering their effect sizes and distributional overlap, as illustrated in Figure 23. Similarly, listeners in our study consistently and reliably used f0 in perception to distinguish between registers. While one could argue on this basis that f0 is the “primary” cue, this could be taken to imply that speakers are in some sense seeking to produce particular f0 targets for high and low registers, as they might in a tone language. Instead, we suspect that what speakers “target” is a general laryngeal setting, rather than specific acoustic values of individual cues.

### 4.4 Vowel quality as an emerging cue to Mon registers?

Previous studies have identified differences in vowel quality across registers, though these differences are only significant for certain vowels (Behr 2018; Thongkum 1987). Specifically, low register vowels tend to be realized with lower F1 and F2 than high register vowels. This has led some researchers to suggest that vowel quality is an emerging cue for register in Burma Mon (Behr 2018). However, others argue that these differences are minor, particularly when considering the influence of f0 and F3 (Abramson et al. 2015). An important question, then, is whether our findings can help clarify if vowel quality should indeed be considered an emerging cue for Mon register.

**Figure 26:**
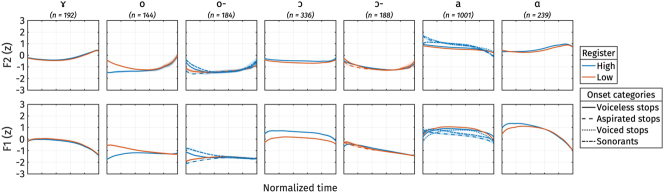
Normalized F2 (top) and normalized F1 (bottom) trajectories for different registers by onset categories. Ribbons represent the standard error of means.

In this paper, we observed significant differences in vowel quality across registers only for three vowels: /o/ in closed syllables, /ɔ/ in closed syllables, and /ɑ/. However, each vowel exhibited effects in different directions, as summarized in Table 10. We did not observe any register-induced diphthongization of these vowels, contrary to what has been reported by Behr (2018). The observed differences in formant frequencies at the vowel onset, as shown in the mean raw trajectories in Figure 26, can be attributed to the imbalanced distribution of onset place of articulation in the wordlist (see Appendix A).

**Table 10:** Vowel quality observed across registers for vowels showing significant differences.

Vowel	High register	Low register
/o/	higher, more back	lower, more front
/ɔ/	lower, more front	higher, more back
/ɑ/	lower, more front	higher, more back

As shown in Table 10, only the vowels /ɔ/ and /ɑ/ follow the pattern observed in previous studies of Mon and align with the typological patterns of register languages summarized in Table 1. This lack of consistency, both among the vowels in our sample and when compared to the broader typology of register languages, suggests that the vowel quality differences we observed may not serve as straightforward cues to register contrast. Instead, these differences could be influenced by other factors, which may interact with the register distinction in complex ways. In future work, we hope to conduct a more systematic investigation of the role of vowel quality using a corpus with a more complete and balanced set of vowels.

## 5 Conclusions

This study investigated both the production and perception of the register contrast in Mon. For the production analysis, we examined acoustic measurements and Open Quotient of EGG signals using Functional Principal Component Analysis (FPCA), a novel approach in the study of register contrasts. This method enabled us to more comprehensively capture the dynamic and multidimensional nature of the acoustic properties of register. In our perception experiment, we systematically manipulated pitch, voice quality, and vowel quality to determine the relative weighting of these cues to register.

Our findings reveal that fundamental frequency (f0) and voice quality are the main correlates in both the production and perception of Mon registers. Vowel quality, while showing some variation across registers, does not exhibit a consistent or systematic pattern across all vowels, suggesting it plays a more limited role. Additionally, we found that a single PC score modulated both f0 and voice quality, consistent with these being acoustic consequences of a single shared laryngeal setting. Finally, while our results suggest that f0 is a generally a more robust perceptual cue to register in Mon, we also see evidence that listeners are sensitive to vowel and voice quality, particularly when f0 is ambiguous.

These findings contribute to our understanding of the acoustic bases of register contrasts in Mon and suggest potential areas for further investigation, particularly in exploring the interaction between f0 and voice quality and their relative stability across different contexts. Future research could also benefit from a more comprehensive analysis of vowel quality, considering a more balanced set of vowel tokens.

## Figures and Tables

**Figure 24:**
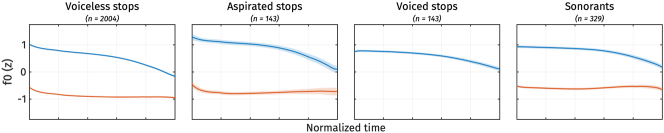
Normalized f0 trajectories for different registers by onset categories. Ribbons represent the standard error of means.

**Figure 25:**
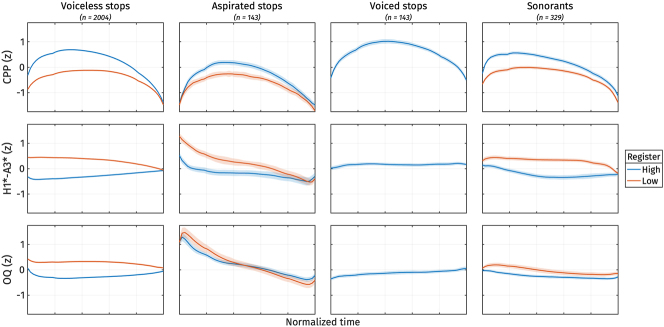
Normalized CPP (top), normalized H1*-A3* (middle), and normalized OQ (bottom) trajectories for different registers by onset categories. Ribbons represent the standard error of means.

**Figure 27:**
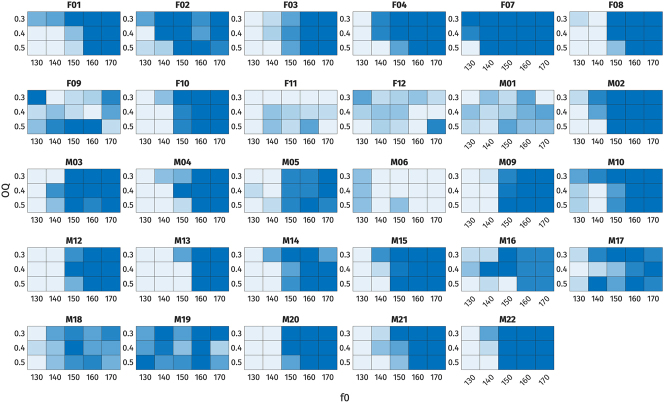
Heat plots of high register responses, shown plotted across each combination of f0 and OQ for each listener.

**Figure 28:**
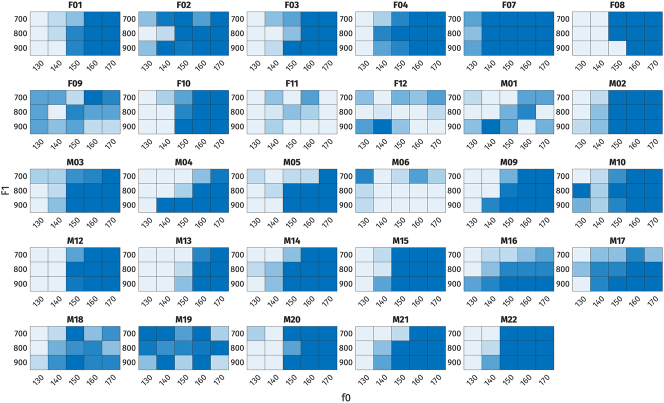
Heat plots of high register responses, shown plotted across each combination of f0 and F1 for each listener.

**Figure 29:**
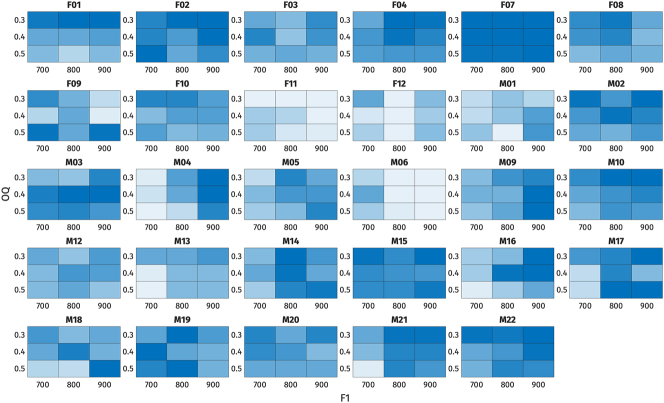
Heat plots of high register responses, shown plotted across each combination of F1 and OQ for each listener.

## Appendix A: Wordlist

**Table:**

IPA	Orthography	Romanization	Register	Gloss
da		kḋā	high	shallow, unimportant
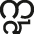
daiŋ		kḋāṅ	high	steel
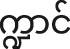
daŋ		kḋaṅ	high	expensive
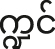
ka		kā	high	car
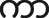
kah		kaḣ	high	to scrape off, to shave
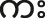
kan		kān	high	flax, jute
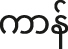
kat		skāt	high	strong, harsh
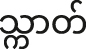
kaʔ		ka	high	fish (general term)
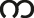
kok		kok	high	to call, to name, to summon
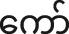
klan		klān	high	to lick
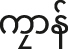
klaʔ		kloʔ	high	to cross (street, river)
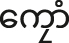
klɤŋ		kluṅ	high	to come
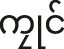
kɔ		kaw	high	to snap, to break; to carry sth. on shoulders
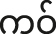
kom		koṁ	high	to assemble, come together
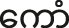
kɑ̤n		gān	low	to push
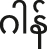
kɑ̤ŋ		guiṅ	low	to grasp, to keep, to take, to take away, to put away
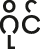
wa̤t.ka̤t		wātgāt	low	very difficult
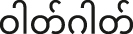
kla̤n		glān	low	naughty
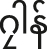
klɤ̤ŋ		gluṅ	low	boat
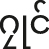
kɔ̤h		gah	low	Particle marking noun and nominal phrase
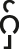
kɔ̤k		gaṁk	low	to feel cold, to shiver, to be cold to the touch
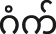
kɔ̤n		sgan	low	to hold (with the hand)
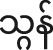
kɔ̤ŋ		gaṁṅ	low	bold, brave
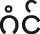
ko̤		gow	low	handsome
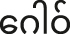
kʰa		khā	high	amaranth leaves
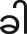
loiŋ		liṅ	high	firefly /(kə)maʔ lak loiŋ/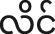
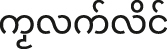
lo̤iŋ		liṅ	low	hire, rent
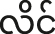
na		ṅā	high	to take away
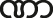
nɛ̤a		gnāw	low	stomach, bowels
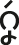
no		tnow	high	lineage, affiliation
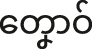
noiŋ		kniṅ	high	cheek; cluster; razor
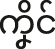
no̤iŋ		gniṅ	low	waist
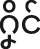
ta		tā	high	toddy palm
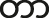
tah		taḣ	high	to level, to level off
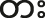
tan		tān	high	to weave, to braid
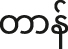
tɑŋ		tuiṅ	high	post; creel (basket)
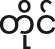
tap		tāp	high	to (arrive in time to) catch
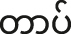
taʔ		ta	high	father (as in /jäj taʔ/ “parents”
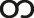
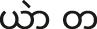
tɑk		tuik	high	building
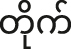
tɔ		taw	high	shaft, rod, handle
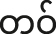
tɔŋ		taṁṅ	high	to pull, draw, lead
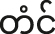
tɤŋ		tuṅ	high	to soak
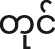
to		tow	high	cotton (plant)
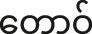
tɑ̤n		dān	low	to donate
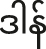
tɑ̤ŋ		duiṅ	low	stork
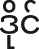
tɑ̤t		dāt	low	to slap
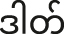
tɑ̤k		duik	low	poor
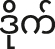
te̤ʔ		deʔ	low	younger sibling
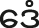
tɔ̤		daw	low	to close (door); cover, hide
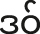
to̤m		doṁ	low	to fall over
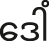
tɔ̤h		dah	low	to be (in a state of)
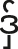
tɔ̤ŋ		daṁṅ	low	to leap (with feet together), to jump
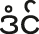
tɤ̤ŋ		duṅ	low	to receive, to accept
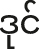
tʰo		thow	high	hornet
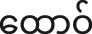
tʰɔ̤		dhaw	low	straight (ahead); dharma
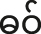

## Appendix C: Linear mixed-effect model outputs

**Table 11:** Summary of linear mixed effect models for PC-scores *s*_1_ and *s*_2_ for f0.

	*s*_1_ model	*s*_2_ model
(Intercept)	−4.80 (0.22)^***^	−0.44 (0.47)
register.low	10.09 (0.36)^***^	0.97 (0.32)^**^
Marginal *R* ^2^	0.60	0.03
Conditional *R* ^2^	0.61	0.50
AIC	11,300.82	8,306.04
BIC	11,334.44	8,339.66
Log likelihood	−5,644.41	−4,147.02
Num. obs.	2,004	2,004
Num. groups: subj	17	17
Var: subj (intercept)	0.53	3.64
Var: subj register.low	1.68	1.64
Cov: subj (intercept) register.low	−0.94	−1.28
Var: residual	16.17	3.46

^***^ *p* < 0.001; ^**^ *p* < 0.01; ^*^ *p* < 0.05.

**Table 12:** Summary of linear mixed effect models for PC-scores *s*_1_ and *s*_2_ for voice quality measures (CPP, H1*-A3*, and open quotient).

	*s*_1_ model	*s*_2_ model
(Intercept)	−3.39 (0.47)^***^	−1.11 (0.37)^**^
register.low	7.12 (0.98)^***^	2.33 (0.78)^**^
Marginal *R* ^2^	0.30	0.05
Conditional *R* ^2^	0.39	0.14
AIC	12,244.98	11,958.43
BIC	12,278.60	11,992.04
Log likelihood	−6,116.49	−5,973.21
Num. obs.	2,004	2,004
Num. groups: subject	17	17
Var: subject (intercept)	3.28	2.02
Var: subject register.low	15.42	9.42
Cov: subject (intercept) register.low	−6.88	−4.30
Var: residual	25.43	22.18

^***^ *p* < 0.001; ^**^ *p* < 0.01; ^*^ *p* < 0.05.

**Table 13:** Summary of linear mixed effect models for PC-scores *s*_1_ and *s*_2_ for vowel quality measures (F1 and F2).

	*s*_1_ model	*s*_2_ model
(Intercept)	−6.31 (0.15)^***^	−0.29 (0.13)^*^
register.low	−0.16 (0.29)	1.02 (0.25)^***^
vowelɑ	0.14 (0.30)	0.10 (0.30)
vowelɤ	9.59 (0.30)^***^	−1.13 (0.30)^***^
vowelo	19.59 (0.30)^***^	−0.20 (0.30)
vowelo-	21.49 (0.40)^***^	−1.20 (0.41)^**^
vowelɔ	6.37 (0.40)^***^	4.65 (0.41)^***^
vowelɔ-	16.65 (0.30)^***^	0.90 (0.30)^**^
register.low:vowelɑ	1.11 (0.41)^**^	−0.90 (0.42)^*^
register.low:vowelɤ	−0.04 (0.44)	−0.39 (0.45)
register.low:vowelo	−2.53 (0.52)^***^	−0.72 (0.52)
register.low:vowelo-	0.29 (0.59)	−1.02 (0.60)
register.low:vowelɔ	3.43 (0.46)^***^	−2.67 (0.47)^***^
register.low:vowelɔ-	0.32 (0.53)	−1.20 (0.54)^*^
Marginal *R* ^2^	0.88	0.20
Conditional *R* ^2^	0.88	0.21
AIC	9,213.59	9,271.92
BIC	9,313.56	9,371.89
Log likelihood	−4,588.79	−4,617.96
Num. obs.	1,908	1,908
Num. groups: subj	17	17
Var: subj (intercept)	0.14	0.03
Var: subj register.low	0.67	0.25
Cov: subj (intercept) register.low	−0.31	−0.09
Var: residual	7.10	7.36

^***^ *p* < 0.001; ^**^ *p* < 0.01; ^*^ *p* < 0.05.

**Table 14:** Summary of linear mixed effect models for PC-scores *s*_1_ and *s*_2_ for f0 and voice quality measures (CPP, H1*-A3*, and open quotient).

	*s*_1_ model	*s*_2_ model
(Intercept)	−5.92 (0.35)^***^	−0.73 (0.44)
register.low	12.44 (0.76)^***^	1.54 (0.85)
Marginal *R* ^2^	0.59	0.02
Conditional *R* ^2^	0.62	0.13
AIC	12,171.46	12,190.26
BIC	12,205.08	12,223.88
Log likelihood	−6,079.73	−6,089.13
Num. obs.	2,004	2,004
Num. groups: subject	17	17
Var: subject (intercept)	1.70	2.93
Var: subject register.low	9.03	11.49
Cov: subject (intercept) register.low	−3.88	−5.67
Var: residual	24.72	24.84

^***^ *p* < 0.001; ^**^ *p* < 0.01; ^*^ *p* < 0.05.
